# The draft genome of the specialist flea beetle *Altica viridicyanea* (Coleoptera: Chrysomelidae)

**DOI:** 10.1186/s12864-021-07558-6

**Published:** 2021-04-07

**Authors:** Huai-Jun Xue, Yi-Wei Niu, Kari A. Segraves, Rui-E Nie, Ya-Jing Hao, Li-Li Zhang, Xin-Chao Cheng, Xue-Wen Zhang, Wen-Zhu Li, Run-Sheng Chen, Xing-Ke Yang

**Affiliations:** 1 Institute of Zoology, Chinese Academy of Sciences, Beijing, 100101 China; 2 College of Life Sciences, Nankai University, Tianjin, 300071 China; 3 Institute of Biophysics, Chinese Academy of Sciences, Beijing, 100101 China; 4 University of Chinese Academy of Sciences, Beijing, 100049 China; 5 Department of Biology, Syracuse University, 107 College Place, Syracuse, NY 13244 USA; 6 Archbold Biological Station, 123 Main Drive, Venus, FL 33960 USA; 7 Biomarker Technologies Corporation, Floor 8, Shunjie Building, 12 Fuqian Road, Nanfaxin Town, Shunyi District, Beijing, 101300 China

**Keywords:** *Altica*, Genome, Annotation, Host plant adaption, Chemosensory, Detoxification

## Abstract

**Background:**

*Altica* (Coleoptera: Chrysomelidae) is a highly diverse and taxonomically challenging flea beetle genus that has been used to address questions related to host plant specialization, reproductive isolation, and ecological speciation. To further evolutionary studies in this interesting group, here we present a draft genome of a representative specialist, *Altica viridicyanea*, the first Alticinae genome reported thus far.

**Results:**

The genome is 864.8 Mb and consists of 4490 scaffolds with a N50 size of 557 kb, which covered 98.6% complete and 0.4% partial insect Benchmarking Universal Single-Copy Orthologs. Repetitive sequences accounted for 62.9% of the assembly, and a total of 17,730 protein-coding gene models and 2462 non-coding RNA models were predicted. To provide insight into host plant specialization of this monophagous species, we examined the key gene families involved in chemosensation, detoxification of plant secondary chemistry, and plant cell wall-degradation.

**Conclusions:**

The genome assembled in this work provides an important resource for further studies on host plant adaptation and functionally affiliated genes. Moreover, this work also opens the way for comparative genomics studies among closely related *Altica* species, which may provide insight into the molecular evolutionary processes that occur during ecological speciation.

**Supplementary Information:**

The online version contains supplementary material available at 10.1186/s12864-021-07558-6.

## Background

The high rate of diversification among host-specific herbivorous insects is thought to result from their shift and specialization to distinct host-plant species, creating conditions that promote reproductive isolation and contribute to the process of speciation [1–3]. One herbivore group that has been used to address questions about host plant specialization, reproductive isolation, and ecological speciation is the leaf beetle genus *Altica* (Coleoptera: Chrysomelidae) [4–10]. This group has undergone rapid divergence that is largely associated with host plant use. For example, studies of three closely related species, *Altica viridicyanea*, *A. cirsicola*, and *A. fragariae*, have demonstrated that although these species are broadly sympatric and quite similar in morphology, they feed on distantly related host plants from different plant families [6]. Consequently, their divergence is likely the result of dietary shifts to unrelated host plants [6]. Further, their close relationship is supported by crossing studies that show that interspecific hybrids can be generated under laboratory conditions [4–6, 8, 10], and phylogenetic analysis indicates that these species diverged over a relatively short period of time. Although *Altica* has been pivotal for understanding the linkages between host plant use and speciation [6, 11–13], the lack of a representative *Altica* genome hinders our ability to more thoroughly investigate the molecular mechanisms underlying the processes of ecological adaptation and diversification within this interesting group.

Key aspects of host plant adaptation and speciation among *Altica* beetles involve both behavioral adaptations to recognize and use the new host plant [14, 15] as well as physiological adaptations that allow them to feed on new plants containing different secondary compounds. These adaptations may involve changes in recognition cues to find the new host plants and new detoxification mechanisms that allow insects to avoid the deleterious effects of defensive chemistry [15, 16]. One aspect of host plant adaptation, then, is the prediction that the gene families involved in the detection of host chemical cues and those involved in xenobiotic detoxification will be key in facilitating successful shifts onto new host plant species. As a result, if we are to understand how host plant adaptation has played a role in speciation of *Altica* beetles, a reference genome would be helpful in making comparisons of candidate gene families involved in the diversification process.

The wealth of genetic and behavioral studies of *A. viridicyanea* makes it an excellent starting point for genomic investigations of host plant adaptation and speciation among *Altica* species. This species is an extreme host specialist of the plant *Geranium nepalense* (Sweet) (Geraniaceae) [4, 5, 7], and reproductive isolation is driven by the presence of species-specific cuticular hydrocarbons (CHC) that determine mating preferences [8, 9]. Studies of F_1_ hybrids involving *A. viridicyanea* have shown that the CHC profiles can also be modified by the beetle’s diet [8, 10]. Consequently, host plant use and mating preferences are intrinsically linked through chemistry.

**Fig. 1:**
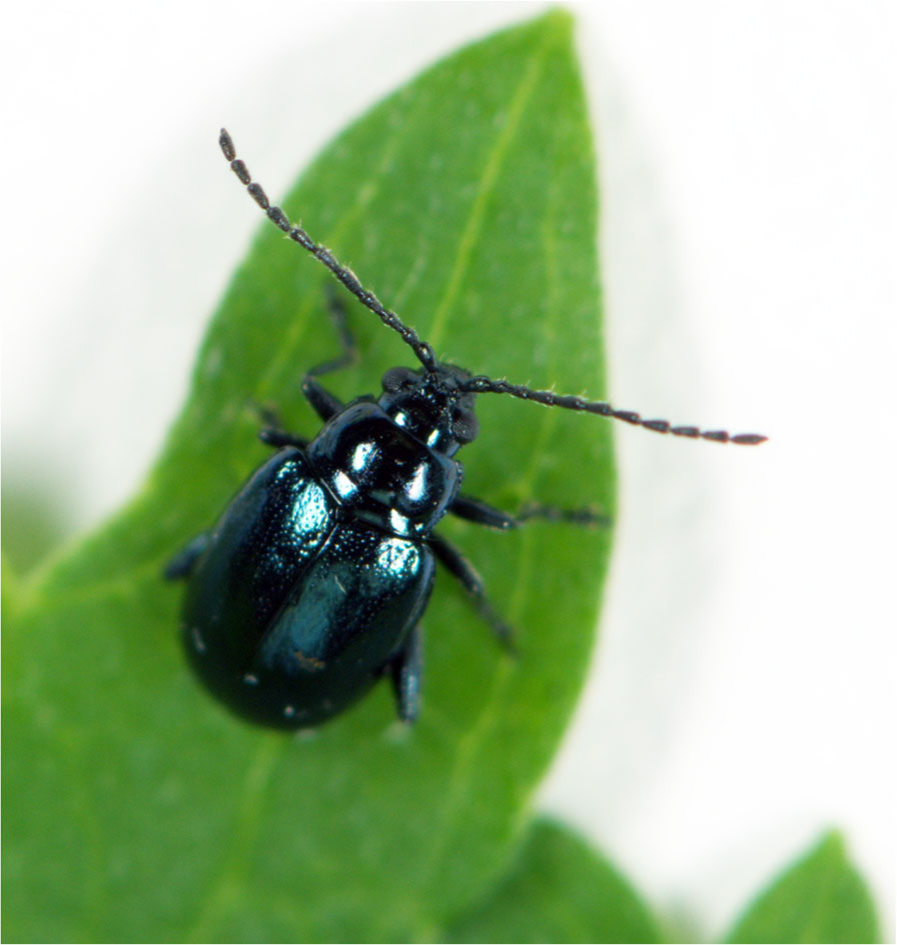
Adult *Altica viridicyanea* (Photographed by Rui-E Nie and Qi-Long Lei)

Here we provide the genome assembly of *A. viridicyanea* (Fig. 1), the first genome of the subfamily Alticinae, and the fifth for the Chrysomelidae. Chrysomelids are a large and highly diverse family of beetles [17], many of which are economically important pests of agricultural crops [18]. The chrysomelid species for which genomes have been assembled exhibit intermediate preferences in host diet and are restricted to feeding on a single plant family (oligophagous). Consequently, the genome for *A. viridicyanea* will add to our genomic resources for a monophagous (restricted to a single host plant species) member of the Chrysomelidae. Furthermore, this assembly will also expand our knowledge of beetles in general as we currently have only 22 published beetle genome assemblies [19–31] (Table S1), a comparatively small number for such a diverse insect group [32–34] (as of May 1, 2020). Finally, the genome assembled here will provide an important resource for further studies on host plant adaptation and functionally affiliated genes.

## Results and discussion

### De novo genome assembly

Flow cytometry revealed that the genome size of *A. viridicyanea* ranged from 836.3 ± 13.8 Mb in females to 795.7 ± 8.3 Mb in males. We generated and assembled 187.3× coverage from Illumina short reads and 72.7× coverage via PacBio long reads from 157 female adults, thus creating a draft genome reference assembly of 864.8 Mb consisting of contig and scaffold N50s of 92.8 kb and 557.2 kb, respectively. The GC content was 31.67%. The size of the *A. viridicyanea* genome was larger than 85% of the currently published beetle genomes (Table S1). The draft genome assembly of *A. viridicyanea* was contained within 17,580 contigs that were assembled into 4490 scaffolds, with the longest scaffold size of 5.6 Mb. Using the reference set of 1658 insect BUSCOs, the genome contains 98.6% complete single-copy orthologs and multi-copy orthologs; using the reference set of 2442 Endopterygota BUSCOs, our genome contains 95.8% complete single-copy orthologs and multi-copy orthologs (Table 1). Together, the results of the above analyses indicate that the genome of *A. viridicyanea* is a robust assembly. Although robust, we note here that the annotated proteins of *A. viridicyanea* were consistently shorter than those from three beetles with relatively high N50 (*Tribolium castaneum*, *Dendroctonus ponderosae*, and *Anoplophora glabripennis*), indicating that there is a potential for gene number inflation caused by the presence of partial genes in the current assembly. The estimated heterozygosity in the Illumina reads was about 0.70% ~ 0.96%, depending on k-mer size (k-mer 17, 19, 21, 23, 25 and 27).

**Table 1:** BUSCO results showing completeness of the *Altica viridicyanea* genome assembly and annotation

	Insect		Endopterygota	
	Counts	Percentage	Counts	Percentage
Complete BUSCOs	1635	98.6%	2340	95.8%
Complete and single-copy BUSCOs	1540	92.9%	2211	90.5%
Complete and duplicated BUSCOs	95	5.7%	129	5.3%
Fragmented BUSCOs	7	0.4%	52	2.1%
Missing BUSCOs	16	1.0%	50	2.1%
Total BUSCO groups searched	1658		2442	

We used these PacBio RNA-seq data to evaluate the genome assembly. Of the 13,550 polished reads, 60.46% could be successfully mapped to the genome. Transcripts that were unmapped or mapped with coverage or identity below the minimum threshold were partitioned into 1177 gene families based on k-mer similarity. Of the 1177 gene families, 121 could be mapped to the assembled genome, and hits to sequences from other species were found for 13 gene families. Blastn revealed that 1032 of the remaining gene families were similar to sequences from plants which may represent DNA contamination by plant material during the DNA isolation step, suggesting that 2 days is not long enough to complete gut clearing in *Altica*. We also analyzed the long reads discarded during QC to identify their origins. Of the 125,390 long reads, 93.27% could be classified, and over 50% of reads were assigned to bacteria. In addition, 37.8% of the reads were assigned to human, indicating contamination during sample and library preparation. These results highlight the importance of checking for genomic contamination during genome assembly.

Furthermore, the addition of HiC or optical mapping data would substantially improve the present genome assembly. Specifically, these additional data would improve the fragmented assembly and would also help resolve the assembly for the sex chromosomes. In the present study, we were limited by the availability of materials; however, future genomic studies in this system will bridge this gap.

### Genome annotation

Prior to gene prediction using the assembled sequences, repeat sequences were identified in the genome of *A. viridicyanea*. The repetitive sequence content was about 62.91% of the assembly, which was similar to that of the cowpea weevil *Callosobruchus maculatus* (64%), lower than that of the ladybird *Propylea japonica* (71.33%), and much higher than that of other beetle species (Table S1). Most of the repetitive sequences were transposable elements. According to a uniform classification system for eukaryotic transposable elements [35], retrotransposons (Class I) accounted for 41.27% whereas DNA transposons (Class II) accounted for 26.24% of the genome (Table 2).

**Table 2:** Composition of repetitive sequences in the *Altica viridicyanea* genome assembly

Repeat type	Number of elements	Length (bp)	Rate (%)
Retrotransposons (transposable element class I)	1,033,903	356,902,348	41.27
DIRS	12,458	7,468,113	0.86
LINE	3,17,300	97,559,643	11.28
LTR/uncertain	45,604	28,406,497	3.28
LTR/Copia	15,362	7,752,670	0.9
LTR/Gypsy	143,535	83,332,210	9.64
LTR or DIRS	91	14,310	0
PLE or LARD	485,362	155,872,484	18.02
SINE	78	80,868	0.01
TRIM	13,553	6,401,760	0.74
Unknown	560	58,144	0.01
DNA transposons (transposable element class II)	803,681	226,943,053	26.24
TIR	550,895	150,574,911	17.41
MITE	10	635	0
Crypton	145,294	43,476,772	5.03
Helitron	29,008	8,403,268	0.97
Maverick	51,701	31,213,827	3.61
Unknown	26,773	5,423,361	0.63
SSR	583	165,863	0.02
Unknown	77,813	23,953,352	2.77
Total	1,848,679	544,002,544	62.91

Notes: *DIRS* dictyostelium intermediate repeat sequence, *LINE* long interspersed nuclear element, *LTR* long terminal repeat, *PLE* penelope-like elements, *SINE* short interspersed nuclear element, *LARD* large retrotransposon derivative elements, *TIR* terminal inverted repeat

To check whether the PacBio reads could span most of the repeats (transposons here), we aligned the PacBio reads to the assembled genome. Focusing on the primary alignment only, there were 89.01% (5,792,616/6,507,752) of reads successfully mapped to the genome. We found that 99.33% (1,913,673/1,926,492) annotated transposons are fully covered by at least one read. Of these regions fully spanned by PacBio reads, the longest one was 29,177 bp. The result shows the length distribution of repeats fully covered and repeats not fully covered by reads. It is clear that repeats fully covered by reads are significantly shorter than those repeats not fully covered. So, we suggested longer reads could help to resolve these regions.

The integration of de novo, RNA-seq-based and homology-based gene prediction methods identified 17,730 protein-coding genes in *A. viridicyanea* (Table 3, Fig. S1), a number slightly less than the average of beetle species with available genomes (~ 18,600 genes on average, Table S1). In total, 16,625 genes were assigned to putative functions, accounting for approximately 93.77% of the predicted genes (Table S2), and 750 putative pseudogenes were identified (Table S3). There were 2462 non-coding RNA models identified, including 45 miRNAs, 1093 rRNAs, and 1324 tRNAs, corresponding to 32, four and 24 gene families, respectively.

**Table 3:** Statistics of gene prediction of *Altica viridicyanea*

Method	Software	Gene number
*ab initio*	Genscan v1.1.0	15,170
	Augustus v2.4	31,813
	GlimmerHMM v3.0.4	58,872
	GeneID v1.4	14,970
	SNAP v2006-07-28	72,679
homology-based	GeMoMa v1.3.1	
	*Drosophila melanogaster*	9310
	*Tribolium castaneum*	14,234
	*Dendroctonus ponderosae*	13,066
	*Anoplophora glabripennis*	18,274
transcriptome-based	TransDecoder v2.0	73,432
	GeneMarkS-T v5.1	29,645
	PASA v2.0.2	23,200
Integration	EVidenceModeler v1.1.1	17,730

### Phylogenetic analysis

**Fig. 2:**
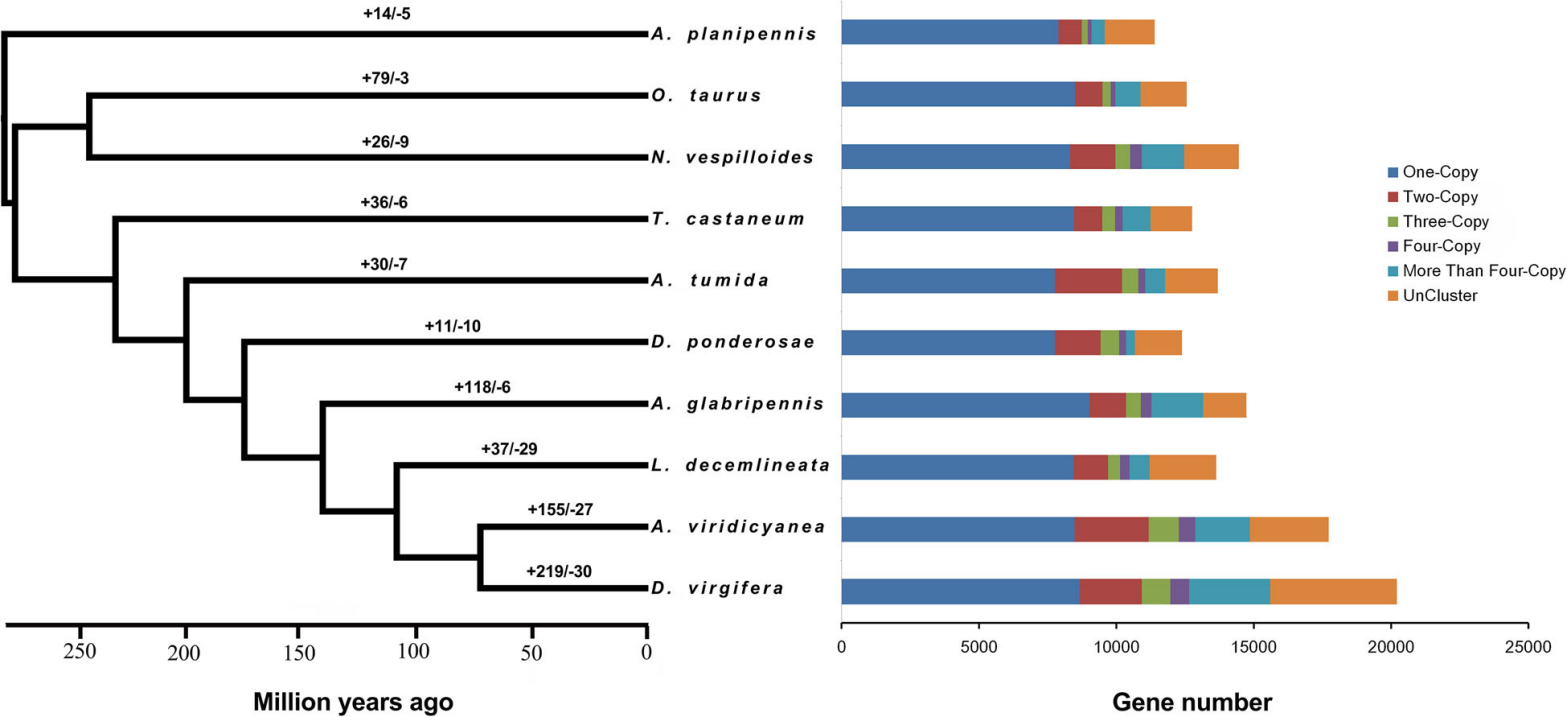
Phylogenetic tree and the proportion of gene family clusters based on ten beetle species. The phylogenetic tree was constructed based on 1751 single-copy orthologs shared among ten beetle species. All nodes have 100% bootstrap support. Branches are labeled with the number of gene family expansions (+) and contractions (−) that occurred on that lineage. These genes were categorized into five groups: one-copy (single copy orthologous genes in common gene families); two-copy (two copy orthologous genes in common gene families), three-copy, four-copy and more than four-copy; uncluster (genes that do not cluster to any families)

We estimated the phylogenetic relationships of *A. viridicyanea* samples and an additional nine representative beetle species (*Anoplophora glabripennis*, *Aethina tumida*, *Agrilus planipennis*, *Dendroctonus ponderosae, Diabrotica virgifera virgifera*, *Leptinotarsa decemlineata*, *Nicrophorus vespilloides*, *Onthophagus taurus* and *Tribolium castaneum*). In total, 14,854 orthologs in *A. viridicyanea* clustered with the other nine representative beetle species. We identified 1321 *A. viridicyanea* specific genes, corresponding to 470 gene families, and with the exception of *Diabrotica virgifera virgifera*, this number was much greater than the other representative beetle species included in this analysis (Table S4)*.* The phylogenetic relationships were consistent with the results inferred from large datasets [36–38] based on 1751 conserved single copy orthologs. For example, *A. viridicyanea*, *Diabrotica virgifera virgifera* and *Leptinotarsa decemlineata*, all belonging to chrysomelids, formed a clade, and these species clustered with *Anoplophora glabripennis*, a member of the superfamily Chrysomeloidea (Fig. 2). The estimated divergence time between *A. viridicyanea* and *Diabrotica virgifera virgifera* was about 74.7 million years ago. From this analysis, we also identified 155 gene families that expanded and 27 gene families that contracted along the *A. viridicyanea* lineage (Fig. 2). Some of these gene families were related to chemosensory and detoxification functions.

### Chemosensory gene families

In many herbivorous insects, feeding, mating and oviposition behaviors are mediated by chemical cues [39]. The chemosensory system may also play important roles in speciation of some insects [40–42]. This is likely the case in *A. viridicyanea* as previous work has shown that this highly specialized beetle primarily uses chemical cues to achieve sexual isolation from its sibling species [8]. Furthermore, these contact chemicals also act as a mating signal to discriminate intraspecific variation in sexual maturity [9]. In addition, chemical cues are modified by and likely involved in host plant choice [8, 10]. Consequently, we investigated *A. viridicyanea* gene families known to be involved in chemosensory signaling in insects.

There are at least five gene families involved in the detection of chemicals, including three receptor families, odorant receptors (ORs), gustatory receptors (GRs) and ionotropic receptors (IRs), and two protein binding families, odorant binding proteins (OBPs) and chemosensory proteins (CSPs). These receptor families are usually expressed in insect olfactory sensory neurons and are involved in the detection of a suite of chemicals. For instance, volatile chemicals are detected by ORs [43–45], contact chemicals or carbon dioxide are detected by GRs [46], and nitrogen-containing compounds, acids, and aromatics are identified by IRs [47]. In contrast, the binding protein gene families are highly abundant in the sensillar lymph of insects and usually function as carriers of hydrophobic scent molecules to the receptors [48, 49].

In the genome of *A. viridicyanea*, we identified 173 putative chemosensory genes and two pseudogenes*.* Perhaps not surprisingly, the gene repertoire of the monophagous *A. viridicyanea* was considerably reduced as compared to that of host generalist species such as *T. castaneum* (630 genes plus 103 pseudogenes) and *A. glabripennis* (451 genes plus 65 pseudogenes)*.* Upholding this pattern, *A. viridicyanea* has fewer chemosensory genes than the oligophagous species such as *Dendroctonus ponderosae* (240 genes plus 10 pseudogenes) and *L. decemlineata* (> 300 genes) that specialize on a single family of host plants (Table S5). Yet there are outliers to this trend; *Agrilus planipennis* (132 genes and two pseudogenes) and *Diabrotica virgifera virgifera* (135 genes, but the gene number for IRs is unavailable) are species that are intermediate in host range, but they have fewer chemosensory genes than *A. viridicyanea*. These findings are generally consistent with the hypothesis that chemosensory gene content and host specificity should correlate in phytophagous beetles [50], although there are clearly exceptions to this rule.

**Fig. 3:**
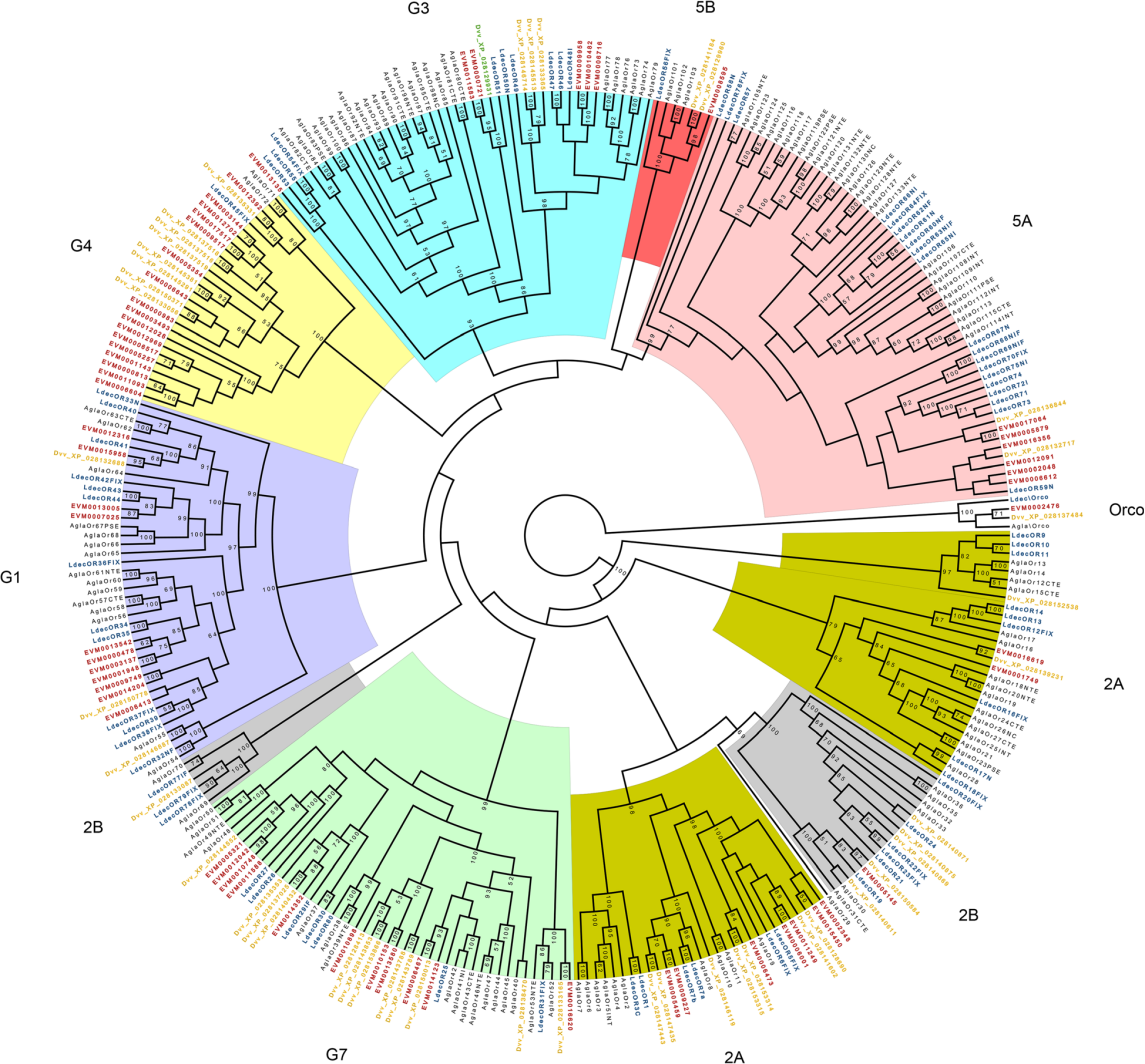
Maximum likelihood cladogram of odorant receptor genes from four beetle species. *Altica viridicyanea* (red labels), *Leptinotarsa decemlineata* (blue labels), *Anoplophora glabripennis* (black labels) and *Diabrotica virgifera virgifera* (Dvv, yellow labels). Node support values lower than 50 are not shown

Insect ORs are proteins with seven transmembrane domains that are involved in the detection of volatile chemicals [44, 51, 52]. The number of ORs in beetle species varies widely from 30 to hundreds of ORs [53]. When we examined the *A. viridicyanea* genome for the presence of ORs, we found a diversity of gene families. There were 63 ORs and one pseudogene (PseudoGene48) that were classified into eight subfamilies: Group 1, 2A, 2B, 3, 4, 5A, 5B and 7 (Fig. 3; Table S5). Following the new OR classification scheme [53], we also identified one highly conserved olfactory co-receptor, Orco, that has been found in other beetle species. Interestingly, we also found a large expansion in *A. viridicyanea* in Group 4 that contained 17 ORs (ten are full length). By comparison, eight Group 4 OR genes have been previously identified in *Diabrotica virgifera virgifera* and no more than four in any other surveyed beetle species [50, 53].

In addition to ORs, we also compared GRs across beetle taxa. Most GRs are expressed in gustatory receptor neurons in taste organs and are involved in contact chemoreception and detection of CO_2_ [46]. We annotated 39 GRs in *A. viridicyanea*, including three conserved candidate CO_2_ receptors, nine candidate sugar receptors, and the remaining were candidate bitter taste receptors. Simple orthology of GRs is generally rare in beetles [50], and not surprisingly, no single-copy orthologs were revealed in the species that we compared. The phylogenetic analysis showed that 2–7 GRs from each of the seven species grouped within the clade of conserved sugar receptors. Additionally, two or three genes from five of the eight species, with the exception of nine genes from *Diabrotica virgifera virgifera*, formed a clade of CO_2_ receptors (Fig. S2).

The number of GRs varied from 10 to 245 in the eleven surveyed beetles (Table S5). Comparisons with *A. viridicyanea* identified as many as 147 GRs in an oligophagous chrysomelid species *Leptinotarsa decemlineata*, and 54 GRs in *Diabrotica virgifera virgifera*, whereas fewer than 20 GRs were annotated in four other chrysomelids (*Colaphellus bowringi*, *Ophraella communa*, *Pyrrhalta aenescens* and *Pyrrhalta maculicollis*). The extremely low numbers of GRs in the latter four species is likely the result of differences in data collection—those species only had transcriptomic data available, and that approach generally does not describe the full complement of chemosensory genes. For example, a study in the longhorn beetle *Anoplophora glabripennis* found 11 GRs when using transcriptomic data, however, genomic data revealed 234 GRs [34, 54].

**Fig. 4:**
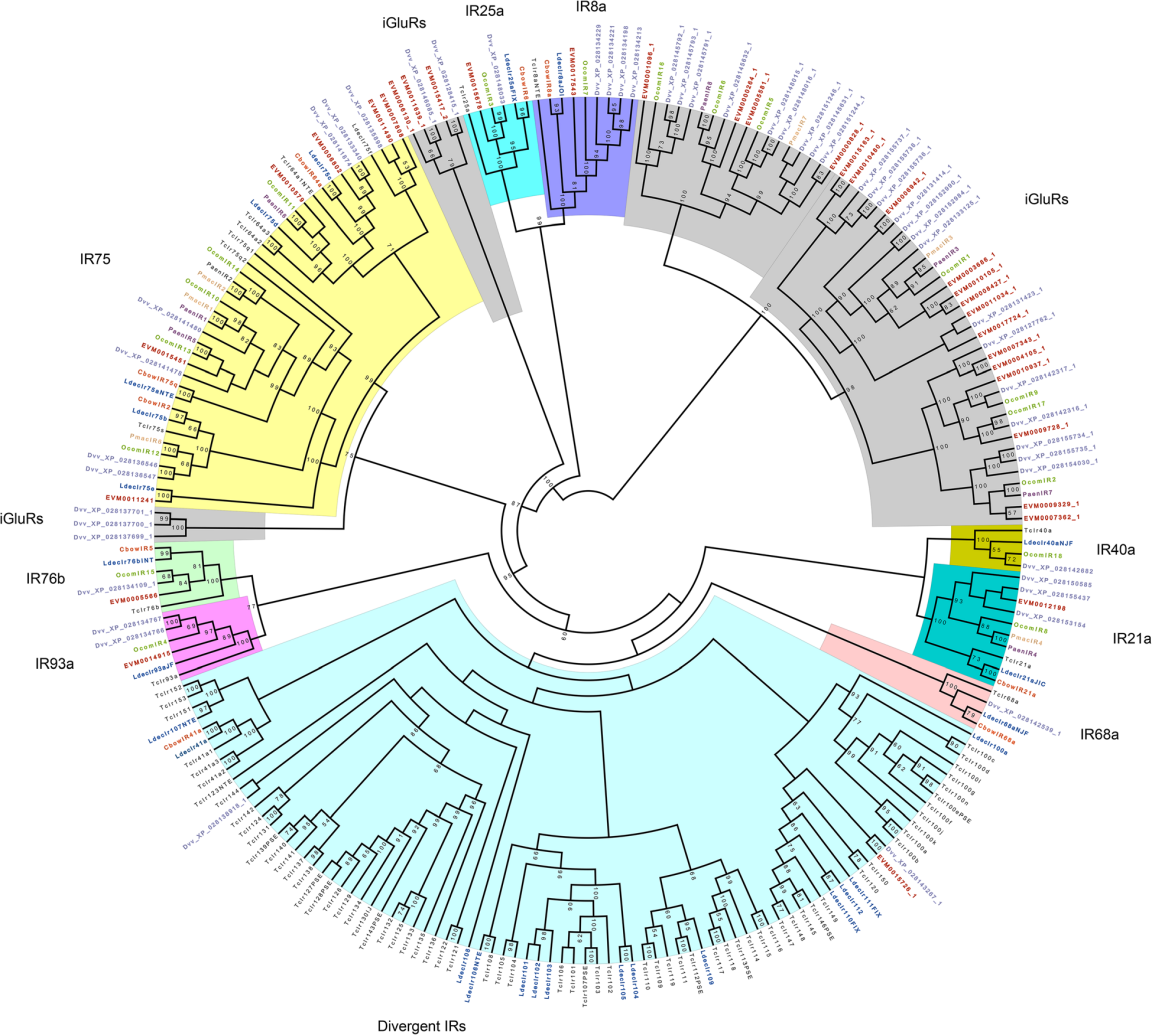
Maximum likelihood cladogram of ionotropic receptor and ionotropic glutamate receptors genes from eight beetle species. *Altica viridicyanea* (red labels), *Ophraella communa* (Ocom, green labels), *Leptinotarsa decemlineata* (Ldec, blue labels), *Colaphellus bowringi* (Cbow, orange labels), *Pyrrhalta aenescens* (Paen, purple labels), *Pyrrhalta maculicollis* (Pmac, yellow labels), *Tribolium castaneum* (Tc, black labels) and *Diabrotica virgifera virgifera* (Dvv, purple gray lable). Node support values lower than 50 are not shown

The next chemosensory receptor group that we examined was the IRs, a conserved family that evolved from a family of synaptic ligand-gated ion channels, ionotropic glutamate receptors (iGluRs) [47, 55, 56]. In insects, the IRs include two groups: the conserved “antennal IRs” that have an olfactory function, and the species-specific “divergent IRs” which are candidate gustatory receptors [57]. Our genome annotations revealed 12 ionotropic receptors (IRs). Only the members of the conserved antennal IR21a group were identified in all eight of the beetle species that we surveyed, whereas the clades IR25a and IR76b were formed by single-copy orthologs from six species, excluding *P. aenescens* and *P. maculicollis* (transcriptomic data are available for both of these species); clade IR8a was also formed by single-copy orthologs from the same six species; however, there were four copies from *Diabrotica virgifera virgifera* (Fig. 4). Furthermore, IRs from all eight species fell within the well-supported non-single-copy IR75 clade (Fig. 4). Compared to other groups, IRs show a contraction in Galerucinae and Alticinae, two closely related subfamilies of Chrysomelidae [58] (*Colaphellus bowringi*, *Chrysomela lapponica*, *O. communa*, *P. aenescens*, *P. maculicollis*, *Diabrotica virgifera virgifera* and *A. viridicyanea*; Table S5).

Finally, we examined the protein binding gene families. OBPs and CSPs are generally regarded as carriers of pheromones and odorants in insect chemoreception, and a multitude of additional functions have also been suggested such as carrying semiochemicals and visual pigments, promoting development and regeneration, and digesting insoluble nutrients [59]. OBPs are small, soluble proteins with six conserved cysteines [48]. Although the detailed mechanisms remain unclear [60], it is believed that OBPs deliver hydrophobic molecules to the receptors [48]. In *A. viridicyanea*, we annotated 48 putative OBP genes and one pseudogene (PseudoGene855). Among these, 34 genes belonged to the Minus C OBPs. We found only one clade of classic OBPs, i.e., Classic VIII, which include single-copy orthologs from each of the eight species in the analysis. In clade IX, two copies from *Diabrotica virgifera virgifera* clustered with single-copy orthologs from seven other species. In clade VII, two copies from *Dendroctonus ponderosae* clustered with single-copy orthologs from other seven species, whereas in Clade X two copies from *Dendroctonus ponderosae* and three copies from *Diabrotica virgifera virgifera* clustered with single-copy orthologs from six other species. Clades I and IV were formed by single-copy orthologs from seven species except for *Diabrotica virgifera virgifera*. The clades of Classic II, III, V and VI were formed by orthologs from 5 to 7 species (Fig. S3). Plus-C OBPs were not found in *A. viridicyanea*, and are also absent in the *Pyrrhalta* species and *Diabrotica virgifera virgifera* that belong to the “Galerucinae+Alticinae” taxonomic group*.*

CSPs are characterized by the presence of four cysteines that form two disulfide bridges [61]. We annotated 12 CSP genes in *A. viridicyanea*. The phylogenetic analysis revealed that only one clade (clade 1) was formed by single-copy orthologs from the eight species surveyed. Clades 2–7 were formed by single-copy orthologs from 5 to 7 beetle species. In these lineages, the absence of IRs from transcriptomic sources (e.g., *P. aenescens*, *P. maculicollis* and *O. communa*) was more common whereas the orthologs of *A. viridicyanea* also lacked members of clade 5 (Fig. S4).

Similar to previous work on GRs, transcriptomes often fail to describe the full set of chemosensory genes due to low expression, spatiotemporal variation in expression, or shallow sequencing depth. For instance, 106 chemosensory genes were detected in *Anoplophora glabripennis* using transcriptomic sequencing [54] whereas more than 500 chemosensory genes (65 pseudogenes included) were annotated from its genome [50].

### Detoxification supergene families

Novel plant secondary compounds often present a challenge for herbivorous insects, and physiological adaptation to novel plant secondary metabolites is a key problem. The detoxification and metabolism of most xenobiotics occurs via a common set of detoxification-related enzymes, all of which belong to multigene families [62]. The cytochrome P450s (P450s), carboxyl/cholinesterases (CCEs), and glutathione S-transferases (GSTs) are widely regarded as the major insect gene/enzyme families involved in xenobiotic detoxification [63–65]. In addition, the UDP-glucuronosyltransferases (UGTs) and ATP binding cassette transporters (ABCs) can also play a role in detoxification [66–68]. This diversity of detoxification enzymes is critical for many herbivorous insects [16, 69] as their diets often contain a suite of plant chemicals that can be toxic, reduce palatability, or slow development time.

The host plant of *A. viridicyanea* is *Geranium nepalens* which has a number of chemical defenses such as tannins, flavonoids and organic acids [70]. As a strict specialist, then, *A. viridicyanea* likely has adaptations that allow them to detoxify these chemicals. Indeed, we annotated 225 detoxification enzymes spanning all three families (101 P450s, 97 CCEs and 27 GSTs). Expansion and contraction of these gene families are considered important in adaptive phenotypic diversification [71]. Furthermore, meta-analyses have established that the size of the P450, CCE and GST gene families are correlated with insect diet breadth [64, 65, 72]. In contrast with these studies, we showed that although *A. viridicyanea* has a greater number of detoxifying genes than that of the closely-related oligophagous *Leptinotarsa decemlineata* (197 genes) [19, 65], it has fewer detoxifying genes than generalist *T. castaneum* (275 genes) [24, 73].

**Fig. 5:**
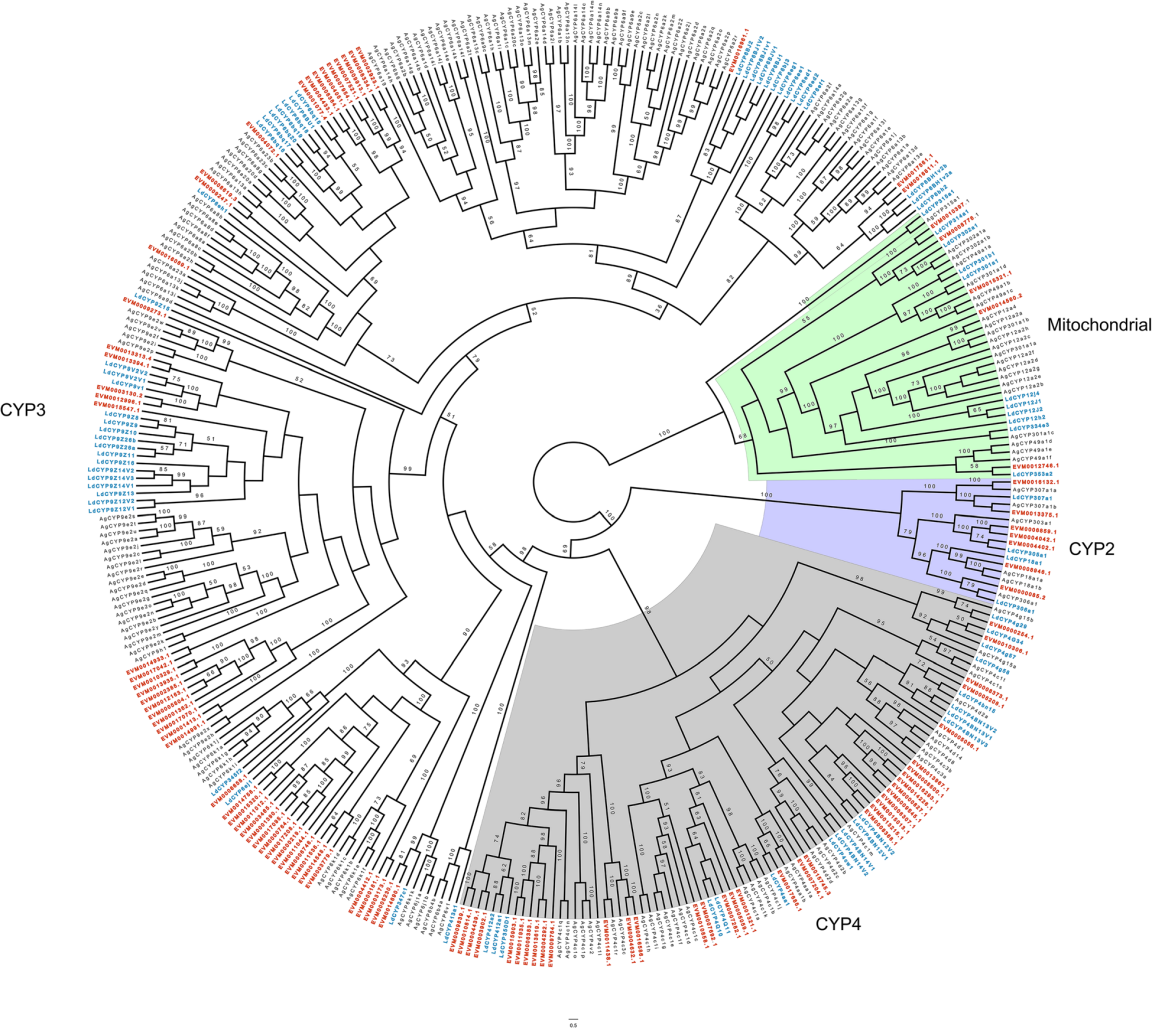
Maximum likelihood cladogram of cytochrome P450s from three beetle species. *Altica viridicyanea* (red labels), *Leptinotarsa decemlineata* (Ld, blue labels) and *Anoplophora glabripennis* (Ag, black labels). Node support values lower than 50 are not shown

Insect cytochrome P450 proteins are important in both xenobiotic detoxification and synthesis and degradation of endogenous molecules such as ecdysteroids and juvenile hormone [74–76]. In insects, the cytochrome P450 family is divided into four major clades: the mitochondrial P450 clade, the CYP2 clade, the CYP3 clade, and the CYP4 clade [77]. We found 101 P450s in *Altica viridicyanea* spanning all four clades: five in the mitochondrial clade, seven in the CYP2 clade, 53 in CYP3 clade, and 36 in CYP4 clade (Fig. 5, Table S6). We found that a majority of these genes belonged to the CYP6 and CYP9 subfamilies of the CYP3 clade and the CYP4 subfamily of the CYP4 clade (Table S7). These P450 subfamilies are known to be involved in detoxification of plant allelochemicals as well as resistance to pesticides [19, 78–80].

In addition to the cytochrome P450s, the *A. viridicyanea* genome also contained 97 genes encoding putative CCEs (Fig. S5), which is slightly fewer than that of *L. decemlineata* (102)*,* but more than that of the other eight beetle species that were included in the analysis (ranged from 44 to 82) [65]. The dietary/detoxification group included two clades: coleopteran xenobiotic metabolizing CCE (clade A) and ɑ-esterase type CCEs (clade B) [81]. In *A. viridicyanea*, there is a noteworthy expansion (62 genes) in clade A, whereas we did not identify any genes from Clade D (integument esterase), F (juvenile hormone esterase), or I (unknown function) (Fig. S5; Table S8).

Another group of detoxification enzymes that we examined are the GSTs. GSTs are involved in many cellular physiological activities, such as detoxification of endogenous and xenobiotic compounds, intracellular transport, biosynthesis of hormones and protection against oxidative stress [73, 81]. Insect GSTs are divided into two major groups, the cytosolic and the microsomal GST genes. The cytosolic group is further divided into six classes: Delta, Epsilon, Sigma, Omega, Theta, and Zeta [82]. The Delta and Epsilon classes are thought to be insect-specific [73, 83, 84], and members of the Epsilon subfamily are commonly involved in detoxification of xenobiotics [85]. We detected a total of 27 GST genes in *A. viridicyanea* (Fig. S6; Table S9)*.* Both the total number and the number of detoxification-related Epsilon subfamily in *A. viridicyanea* were lower than that of most beetles [65](Table S9).

UDP-glycosyltransferases (UGT) catalyze the conjugation of a range of diverse small lipophilic compounds with sugars to produce glycosides, playing an important role in the detoxification of xenobiotics and in the regulation of endobiotics in insects [66]. From 17 (*Oryctes borbonicus*) to 65 (*Anoplophora glabripennis*) UGTs were identified in the nine beetle species surveyed [3, 65]. Currently, the largest repertoire of UGTs in beetles was found in the polyphagous longhorn beetle *Anoplophora glabripennis*, with 65 putative UGT genes and 7 pseudogenes [34]. The expansion of UGTs in *A. glabripennis* is thought to be related to its ability to feed on a broad range of host plants [34]. In line with this, we annotated 32 UGTs in the *A. viridicyanea* genome. A number of UGT50s were identified in this species, which has been suggested as the most conserved UGT in insects [66], and we also observed a remarkable expansion in the UGT324 family (Fig. S7, Table S10).

**Fig. 6:**
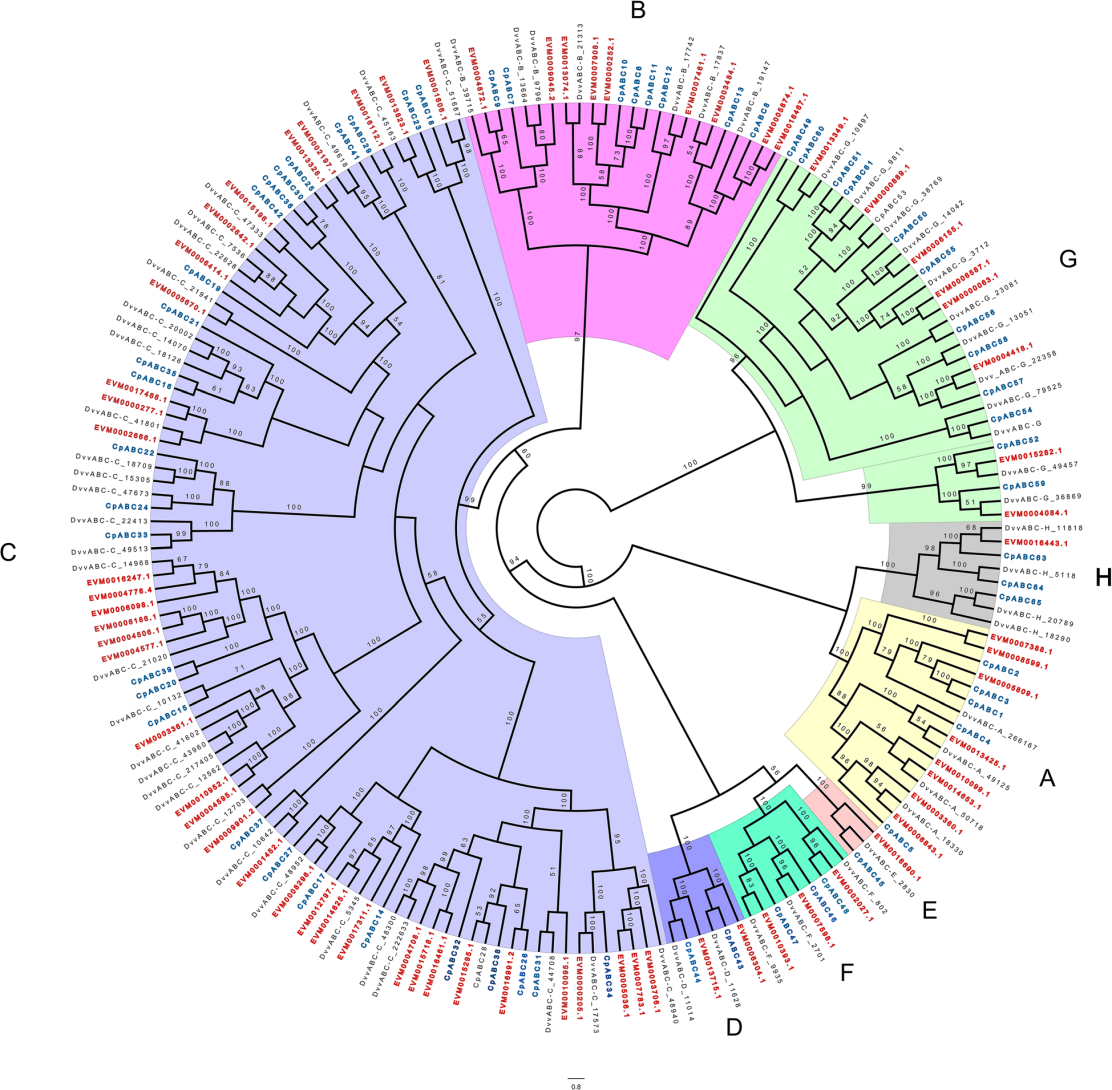
Maximum likelihood cladogram of ATP binding cassette transporters (ABC) from three beetle species. *Altica viridicyanea* (red labels), *Chrysomela populi* (Cp, blue labels) and *Diabrotica virgifera virgifera* (Dvv, black labels). Node support values lower than 50 are not shown

Most ABC proteins engage in active transport of molecules across cell membranes. The ABC transporters are well-known components of various detoxification mechanisms across all phyla [86, 87]. In the present study, we identified 69 putative ABCs in *A. viridicyanea*, belonging to eight subfamilies (A to H). This is a similar number to two specialist species of chrysomelids, *Chrysomela populi* and *Diabrotica virgifera virgifera*, (65 in each based on transcriptomic data) (Table S11). The gene numbers of the conserved subfamilies D, E and F were consistent with other beetles analyzed (Table S11); however, the number of genes in subfamilies B and C in *A. viridicyanea* (46) are the highest among the five species with which we compared (Table S11; Fig. 6). These subfamilies are known to be involved in detoxification processes [62, 88].

### Plant cell wall-degrading enzymes

Early views of insect digestion postulated that insects lack the endogenous enzymes required for plant cell wall (PCW) digestion, and that PCW digestion by insects depended on exogenous enzymes from symbiotic microorganisms [89]. Recent studies, however, have revealed that endogenous PCW degrading enzymes are present in many insects and are important in the digestion of cellulose, hemicelluloses, and pectin in PCW [38, 90]. In fact, these enzymes are likely a key innovation in the adaptive radiation of herbivorous beetles. Some insect PCW-degrading enzymes are also involved in immune-defense responses and detoxification [38, 90].

Beetle-encoded plant cell wall-degrading enzymes are carbohydrate esterases (CE), polysaccharide lyases (PL), and mainly glycoside hydrolases (GH) [38]. In *A. viridicyanea*, we identified 65 putative glycoside hydrolases, including 35 GH1 genes, 10 GH45 genes, two GH48 genes and 18 GH28 genes (Table S12). Genes of GH1 originated anciently in animals and are ubiquitous in beetles. The species of Phytophaga (i.e., Chrysomeloidea and Curculionoidea) examined thus far have a greater number of GH1 genes than *A. viridicyanea*, for example, 228 were found in *Diabrotica undecimpunctata*, 135 in *Mastostethus salvini*, and 136 in *Rhynchitomacerinus kuscheli* [19, 34, 38]. For another ancient and ubiquitous gene family, GH9, there are at least a dozen independent losses in beetles [38]. GH9 was not detected in *A. viridicyanea*, along with 4 of 7 other chrysomelid species (*Callosobruchus maculatus*, *Donacia marginata*, *Diabrotica undecimpunctata* and *Leptinotarsa decemlineata*) [19, 38].

The other plant cell wall-degrading gene families (CE8, PL4, GH32, GH5, GH10, GH43, GH44, GH45, GH48 and GH28) are suggested to be obtained from bacteria and fungi via horizontal gene transfer, and are mainly found in Buprestoidea and Phytophaga, with scattered genes in a few other taxa [38]. In *A. viridicyanea*, in addition to the ubiquitous GH1 genes, three families of PCW-degrading enzymes were identified, including cellulose degrading GH45 and GH48, and pectin degrading GH28. These observed gene numbers are similar to that of closely related species in the Chrysomelinae (*Oreina cacaliae* and *Leptinotarsa decemlineata*) and Galerucinae (*Diabrotica undecimpunctata*) [19, 38].

## Conclusions

In the present study, we combined long reads of PacBio with the higher fidelity of the short reads generated with Illumina sequencing. From the genome annotation we found that *A. viridicyanea*, a host specialist herbivore, has a reduced number of chemosensory and detoxification genes as compared to more generalist herbivorous beetles, consistent with the idea that diet breadth should positively correlate with chemosensory and detoxification gene content. Although *A. viridicyanea* had fewer chemosensory and detoxification genes than more polyphagous beetles, we did observe expansions in some gene families that may be related to host plant adaptation. As a result, the genome assembled here provides an important resource for further studies on host plant adaptation and functionally affiliated genes. Additionally, this work will also open the opportunity for comparative genomics studies among closely related *Altica* species that may provide insights into the molecular evolutionary processes that occur during ecological speciation.

## Methods

### Beetles

*Altica viridicyanea* is a highly specialized herbivore of *Geranium nepalense*. They are elongate-ovate beetles with a length of 3–4 mm. The dorsal surface is black with a metallic blue reflection (Fig. 1). Equipped with dilated hind legs, these beetles typically jump in a flea-like fashion to escape predators.

To make genome sequencing easier, we created a laboratory colony by collecting adult *A. viridicyanea* in Changping (40.28′N, 116.05′E), Beijing, China. Adults were maintained in growth chambers held at 16:8 LD and 25 °C and fed with leaves of their host plant, *Geranium nepalense* (Sweet). A subset of these collected adults was used for genome size estimation (see below). We maintained the colony through successive single-pair sibling matings to create third generation lines for whole genome sequencing. When we were ready to sequence the beetles, beetles were starved for 2 days and then killed in liquid nitrogen. The samples were stored at − 80 °C until DNA extraction. In total, we used 157 virgin females for sequencing.

### Genome size estimation

In preparation for whole genome sequencing, we first determined the genome size of *A. viridicyanea*. Genome size was estimated via flow cytometry [91] on four adult males and four adult females. The thoracic muscle of living beetles was dissected with sterilized fine forceps, and cut into small pieces in Galbraith buffer. The ground suspension was filtered through 40-mm nylon mesh (Easystrainer™) to remove cellular debris and the flow-through was collected in a 5-ml round-bottomed tube placed on ice. Propidium iodide was add to samples to a final concentration of 50 mg/ml and stained in the dark at 4 °C for 2 h. Then fluorescence intensity was estimated for each beetle using a superfluid cell sorting system (MoFol XDP, Beckman Coulter Life Sciences). Genome size was calculated by comparing samples to an internal reference subsisting of chicken blood.

### DNA extraction and sequencing

Genomic DNA (gDNA) was extracted from whole beetles using DNeasy Blood & Tissue Kit (Qiagen, Germany) following the manufacturer’s instructions. Samples were first surface sterilized using 75% ethanol and sterile deionized water. The total amount of gDNA was measured using QubitFluorometer (Invitrogen), and the integrity of the gDNA was verified on an agarose gel that had reference lanes containing high molecular weight ladders (GeneRuler High Range DNA Ladder and D2000 DNA Ladder). From these extractions, seven Illumina sequencing libraries were prepared, with insert sizes and genome coverage of 270 bp (55.5×), 500 bp (29.4×), 800 bp (19.0×), 3 kb (12.5×), 5 kb (two libraries, 49.7×), to 10 kb (21.1×) (Table S13). The libraries were sequenced with 150 bp paired-end reads on the Illumina HiSeq 2500 platform. We also sequenced two PacBio libraries with 72.7× genome coverage, with N50 read lengths of 9.1 kb (Table S13). The first PacBio DNA library (20 kb) was constructed using the PacBio SMRTbell Template Prep Kit 3.0 (Pacific Biosciences, Menlo Park, CA, USA) and sequenced on a PacBio RS II sequencer with the P6 polymerase/C4 chemistry combination at 1GENE (Zhejiang, China). A total of 16 SMRT Cells were processed and the movie length was 6 h. The second PacBio DNA library (20 kb) was constructed using the SMRTBell template preparation kit 1.0 (PacBio, USA), for which six SMRT Cells were run on the PacBio Sequel instrument at BGI (Guangdong, China) with SequelTM Sequencing Kit 2.1 (PacBio, USA). The movie length was 10 h.

### RNA-seq library construction and sequencing

We produced transcriptomic data to facilitate gene prediction analyses. To obtain more full-length or near full-length gene sequences, we generated sequence data by combining PacBio full-length transcriptome sequencing (8 Gb clean data) and Illumina sequencing (10 Gb clean data). Whole bodies of four males and four females with three libraries (size-selection: 1–2 kb, 2–3 kb and > 3 kb lengths) were used for PacBio sequencing. The PacBio transcriptome libraries were constructed using PacBio SMRTbell Template Prep Kit 3.0 (Pacific Biosciences, Menlo Park, CA, USA) and sequenced on a PacBio RS II sequencer at 1GENE (Zhejiang, China). The sequencing chemistry was P6-C4, and the movie length was 4 h. Five SMRT Cells were processed: one for the 1–2 kb library, two for the 2–3 kb library and two for the > 3 kb library. To enrich for chemosensory genes, heads from 30 females and 30 males were also used for Illumina sequencing.

### Data pre-processing

For Illumina Data, FastQC v0.11.6 (http://www.bioinformatics.babraham.ac.uk/projects/fastqc/) was used to check the quality of the raw reads under default settings, and then TrimGalore v0.4.5 (https://github.com/FelixKrueger/TrimGalore) was used to filter out the adapters and low-quality reads using the parameters “--length 36 -q 20 --trim-n”. Because *A. viridicyanea* are quite small, we used the whole body of adults for sequencing. Although we surface sterilized the insects, the likelihood of microbial contamination from gut contents was high; therefore, we used BBDuk in the BBMap v37.80 (https://sourceforge.net/projects/bbmap) with parameters “ordered = t k = 31” to filter microbial contaminants. To do this, we first built a reference library which included all sequences of archaea, bacteria, fungi, protozoa, and viruses available in RefSeq (https://ftp.ncbi.nlm.nih.gov/genomes/refseq/) (accessed on August 15, 2019). The mitochondrion sequences of *A. viridicyan*ea (GenBank accession numbers: MH477594, MH477596, MH477598 and MH477599) [92] were also included to remove reads originating from the mitochondrial genome. After these filtering steps, the remaining reads were used for downstream genome assembly and analysis.

To simplify the genome assembly process, we also excluded reads originating from microbial sources that were present in the PacBio data. To accomplish this, we mapped all the raw PacBio reads to the above Illumina library using minimap2 v2.12 [93], using the parameters “-x map-pb -a -Q --split-prefix”. To improve the mapping specificity, we included 11 available coleopteran genomes [26] in the reference library. Then we removed the reads that mapped to the microbial sequences.

For the PacBio Iso-Seq data processing, we used the new Iso-Seq3 pipeline (https://github.com/PacificBiosciences/IsoSeq_SA3nUP). First, we first converted the bax.h5 files of each SMRT Cell run to the BAM format using bax2bam v0.0.8 (https://github.com/PacificBiosciences/bax2bam) with default parameters. Circular Consensus Sequence (CCS) calling was then done by ccs v3.4.1 (https://github.com/PacificBiosciences/ccs) under default settings. To remove cDNA primers and obtain full-length reads, we used lima v2.0.0 (https://github.com/pacificbiosciences/barcoding) with parameters “--isoseq --peek-guess”. Next we used “isoseq3 refine --require-polya” to trim the poly (A) tails. Finally, to generate polished, high-quality reads, the SMRT cells were merged and we used “isoseq3 cluster” with parameters “--use-qvs”.

### Heterozygosity estimation

Heterozygosity was estimated using gce v1.0.0 (parameters: -b 1 -H 1 -m 1 -D 8), based on the pooled Illumina reads of the sib-mated females. Before running gce, we first used kmer_freq_hash in the gce package to compute the histogram of kmer frequencies (k-mer from 17 to 25).

### Genome assembly

A hybrid assembly strategy was used to assemble the PacBio and Illumina reads. PacBio reads were first assembled using Flye v2.3.5b [94] with the parameters set to “--genome-size 830 m” to obtain contigs. Then Redundans v0.14a [95], which uses an iterative scaffolding approach by calling SSPACE [96], was used to scaffold the PacBio contigs with the Illumina short reads, with parameters set to “--noreduction”. After that, SSPACE-Long v1.1 [97] was used to further scaffold with PacBio long reads using default settings (Table S14).

In addition, we used these PacBio RNA-seq data to evaluate the genome assembly. We mapped PacBio RNA-seq reads to the genome using minimap2 v2.12, with parameters “-ax splice -uf --secondary=no”. For those unmapped and poorly mapped transcripts (coverage < 0.99 or identity < 0.95), the Cogent pipeline (https://github.com/Magdoll/Cogent) was employed to explore their potential functions. Transcripts were partitioned into gene families based on k-mer similarity. For each gene family, contigs were reconstructed to represent the “union” of all coding bases. To check the potential functions of these transcripts, we used Blastn v2.7.1 [98] against the non-redundant sequence database (ftp://ftp.ncbi.nih.gov/blast/db/FASTA; accessed at November 2020).

To evaluate other potential sources of contamination, we examined the long reads discarded during QC to identify their origins. Kraken2 v2.1.1 was used for fast classification of these long reads, with a standard database downloaded from https://benlangmead.github.io/aws-indexes/k2. The output was uploaded to the pavian (https://fbreitwieser.shinyapps.io/pavian/) web server to obtain a report.

### Estimation of genome completeness

Benchmarking Universal Single-Copy Orthologs (BUSCO v3.0.1) [99] analyses against Insecta (*n* = 1658) and Endopterygota (*n* = 2442) were used to evaluate the integrity and quality of the genome assembly with parameters “-sp tribolium2012 -m geno”.

In addition, to characterize the partial genes in our assembly, we chose three beetle species to compare the length of proteins (*Tribolium castaneum*, *Dendroctonus ponderosae*, and *Anoplophora glabripennis*) since the N50 metric of these genomes was relatively high. We downloaded the latest genome annotations for these beetles from RefSeq (Tcas 5.2, DendPond male_1.0 and Agla 2.0), then Blastp v2.7.1 was employed to compare the sequences of the annotated *A. viridicyanea* proteins with proteins from these beetles. Focusing on only the top hit of each query sequence, we compared the lengths between query sequences and subject sequences.

### Genome annotation

Prior to gene prediction using the assembled sequences, we identified repeat sequences in the genome of *A. viridicyanea*. LTR FINDER v1.05 [100], RepeatScout v1.0.5 [101] and PILER-DF v2.4 [102] were used to construct a library of repetitive sequences based on the *A. viridicyanea* genome. PASTEClassifier v1.0 [103] was used to classify these repeats, and the Repbase database [104] was used to merge them. Finally, RepeatMasker v4.0.5 [105] was used to identify and mask the genomic repeated sequences. All the parameters were set to “default” except RepeatMasker where the parameters were set to “-nolow -no_is -norna -engine wublast”. To check whether the PacBio reads could span most of the repeats (transposons here), we aligned the PacBio reads to the assembled genome using minimap2 v2.12 (parameters: -x map-pb). Then Bedtools v2.26.0 [106] was employed to get the read coverage of each repeat.

We combined the de novo, homology-based, and transcriptome-based predictions to identify protein-coding genes in the *A. viridicyanea* genome. The default settings for Genscan v1.1.0 [107], Augustus v2.4 [108], GlimmerHMM v3.0.4 [109], GeneID v1.4 [110] and SNAP (v2006-07-28) [111] were used to generate the de novo predictions. For the homology-based prediction, protein sequences of *Drosophila melanogaster*, *Tribolium castaneum*, *Dendroctonus ponderosae* and *Anoplophora glabripennis* were downloaded from NCBI and aligned to the assembled *A. viridicyanea* genome with GeMoMa v1.3.1 [112, 113] using default parameters. For transcriptome sequencing-based prediction, we firstly assembled the Illumina short reads into unigenes using Hisat v2.0.4 with parameters “--max-intronlen 20000, --min-intronlen 20” [114] and StringTie v1.3.4 set to default parameters [114]. Second, we combined the unigenes with the PacBio full-length transcripts, then we predicted genes based on these combined sequences with PASA v2.0.2 using the parameters “-align_tools gma, -maxIntronLen 20000” [115]. EVidenceModeler v1.1.1 was used to integrate all of the gene predictions [116]. Finally, to obtain a final gene dataset, PASA v2.0.2 was used for further modification including adding 5′-UTR and 3′-UTRs, obtaining alternative splicing, and extending or shortening, adding or subtracting exons for the EVM-predicted results.

We predicted the non-coding RNAs based on the Rfam v12.1 [117] and miRBase v21.0 [118] databases. Putative microRNAs (miRNAs) and ribosomal RNAs (rRNAs) were predicted using Infernal v1.1 [119], and transfer RNAs (tRNAs) were predicted with tRNAscan- SE v1.3.1 [120]. Based on homology to known protein-coding genes, putative pseudogenes were first searched in the intergenic regions of the *A. viridicyanea* genome using genBlastA v1.0.4 [121]. Then GeneWise v2.4.1 [122] was used to search for premature stop codons or frameshift mutations in those sequences.

The predicted genes were annotated by aligning them to the NCBI non-redundant protein (nr) [123], non-redundant nucleotide (nt) [123], Swissprot [124], TrEMBL [124], KOG [125], and KEGG [126] databases using BLAST v2.2.31 [127] with a maximal e-value of 1e^− 5^. We assigned gene ontology (GO) terms [128] to the genes using the BLAST2GO v2.5 pipeline [129].

### Species phylogenetic analysis

We estimated the phylogenetic relationships of *A. viridicyanea* and an additional nine representative beetle species for which genomic data were available (*Anoplophora glabripennis*, *Aethina tumida*, *Agrilus planipennis*, *Dendroctonus ponderosae, Diabrotica virgifera virgifera*, *Leptinotarsa decemlineata*, *Nicrophorus vespilloides*, *Onthophagus taurus* and *Tribolium castaneum*). We used 1751 conserved single copy orthologs for this analysis. The analysis was implemented in PhyML v3.0 [130] with parameters “-gapRatio 0.5 -badRatio 0.25 -bootstrap 1000”.

We also estimated divergence times among the species using MCMCtree (PAML v4.8 package) (http://abacus.gene.ucl.ac.uk/software/paml.html) [131, 132] with parameters set to a burn-in time of 10,000, the sample number was 100,000, and the sampling frequency was 2. We used the time divergence data in timetree (http://www.timetree.org/) for calibration (*Nicrophorus vespilloides* - *Onthophagus taurus* [245 ~ 296 MYA]; *Dendroctonus ponderosae* - *Anoplophora glabripennis* [149 ~ 240 MYA]). To examine gene family expansion and contraction among species, we used CAFE v4.1 [133] with “lambda -l 0.002” to automatically search for birth and death parameters (λ) of genes.

### Gene family evolution

To provide insights into host plant specialization, we specifically focused on the evolution of chemosensory gene families, chemical detoxification supergene families, and plant cell wall-degrading enzymes. We first conducted multiple sequence alignment using Mafft (online version 7.305) [134] (http://mafft.cbrc.jp/alignment/server/), applying the L-INS-I algorithm. The best-fit models for amino acid sequence evolution were selected using the Akaike Information Criterion (AIC) in Prottest v3.4.2 [135]. (Table S15). Maximum likelihood trees were reconstructed using RaxML v8.2.9 [136] in the CIPRES Science Gateway v3.3 (https://www.phylo.org/) [137], with 1000 non-parametric bootstrap replicates. The resulting tree was viewed and edited with FigTree v1.3.1 [138] and Adobe Illustrator CS5.

## Supplementary Information

**Additional file 1: Figure S1.** Venn diagram indicating the number of protein-coding genes of *A. viridicyanea* based on ab initio, RNA-seq-based and homology-based gene prediction methods. **Figure S2.** Maximum likelihood cladogram of gustatory receptor genes from eight beetle species. *Altica viridicyanea* (red labels), *Ophraella communa* (Ocom, dark blue labels), *Colaphellus bowringi* (Cbow, green labels), *Pyrrhalta aenescens* (Paen, purple labels), *Pyrrhalta maculicollis* (Pmac, yellow labels), *Dendroctonus ponderosae* (Dpon, pale blue labels), *Leptinotarsa decemlineata* (Ldec, black labels) and *Diabrotica virgifera virgifera* (Dvv, grey labels). Node support values lower than 50 are not shown. **Figure S3.** Maximum likelihood cladogram of odorant binding proteins from eight beetle species. *Altica viridicyanea* (red labels), *Ophraella communa* (Ocom, yellow labels), *Leptinotarsa decemlineata* (Ldec, green labels), *Colaphellus bowringi* (Cb, pale blue labels), *Pyrrhalta aenescens* (Paen, dark blue labels), *Pyrrhalta maculicollis* (Pmac, orange labels), *Dendroctonus ponderosae* (Dpon, black labels) and *Diabrotica virgifera virgifera* (Dvv, gray lables). Node support values lower than 50 are not shown. **Figure S4.** Maximum likelihood cladogram of chemosensory proteins from eight beetle species. *Altica viridicyanea* (red labels), *Ophraella communa* (Ocom, pale blue labels), *Colaphellus bowringi* (Cbow, purple labels), *Pyrrhalta aenescens* (Paen, orange labels), *Pyrrhalta maculicollis* (Pmac, dark blue labels), *Dendroctonus ponderosae* (Dpon, yellow labels), *Anoplophora glabripennis* (Agla, green labels) and *Tribolium castaneum* (Tcas, black labels). Node support values lower than 50 are not shown. **Figure S5.** Maximum likelihood cladogram of carboxyl/cholinesterases from four beetle species. *Altica viridicyanea* (red labels), *Leptinotarsa decemlineata* (Ldec, yellow labels), *Aethina tumida* (At, blue labels) and *Tribolium castaneum* (Tc, black labels). Node support values lower than 50 are not shown. **Figure S6.** Maximum likelihood cladogram of glutathione S-transferases from four beetle species. *Altica viridicyanea* (red labels), *Leptinotarsa decemlineata* (Ld, blue labels), *Aethina tumida* (Atum, yellow labels) and *Tribolium castaneum* (Tc, black labels). Node support values lower than 50 are not shown. **Figure S7.** Maximum likelihood cladogram of UDP-glucuronosyltransferases from three beetle species. *Altica viridicyanea* (red labels), *Anoplophora glabripennis* (Agla, blue labels) and *Tribolium castaneum* (Tcas, black labels). Node support values lower than 50 are not shown.

**Additional file 2: Table S1.** Published beetle genomes. **Table S2.** Statistics of gene annotation for *Altica viridicyanea* sourced from different databases. **Table S3.** Pseudogenes identified in the genome of *Altica viridicyanea.* **Table S4.** Gene family classification for the beetle species used in the present study. The incomplete genes were excluded from the analysis. **Table S5.** Comparison of number of chemosensory functional genes (+pseudogenes) in *Altica viridicyanea* and other beetle species. **Table S6.** Comparison of the number of CYP450 genes in *Altica viridicyanea* and other beetle species. **Table S7.** Cytochrome P450 genes in *Altica viridicyanea*. **Table S8.** Comparison of number of carboxyl/cholinesterases (CCEs) in *Altica viridicyanea* and other beetle species. **Table S9.** Comparison of the number of glutathione S-transferases (GSTs) in *Altica viridicyanea* and other beetle species. **Table S10.** UDP-glucuronosyltransferases (UGTs) (+pseudogenes) in *Altica viridicyanea* and other beetle species. **Table S11.** Comparison of the number of ABC genes in *Altica viridicyanea* and other beetle species. **Table S12.** Plant cell wall degrading enzymes identified in *Altica viridicyanea* and other beetle species. **Table S13.** Summary of library construction and sequencing of *Altica viridicyanea*. **Table S14.** Summary of de novo assemblies of the *Altica viridicyanea* draft genome. **Table S15.** The best-fit models for amino acid sequence evolution were selected using the Akaike Information Criterion (AIC) in Prottest v3.4.2.

## Data Availability

The raw sequence data reported in this paper are available at the Genome Sequence Archive [139] in National Genomics Data Center [140] (https://bigd.big.ac.cn/gsa; accession number: CRA002741). And the whole genome sequence data have been deposited in the Genome Warehouse in the National Genomics Data Center [140] (https://bigd.big.ac.cn/gwh; accession number: GWHAMMQ00000000).

## Abbreviations

ABC: ATP binding cassette transporter
bp: Base pairs
BUSCO: Benchmarking Universal Single-Copy Orthologs
CCEs: Carboxyl/cholinesterase
CE: Carbohydrate esterase
CSP: Chemosensory protein
Gb: Gigabase pairs
GH: Glycoside hydrolase
GR: Gustatory receptor
GST: Glutathione S-transferase
IR: Ionotropic receptor
kb: Kilobase pairs
LINE: Long interspersed nuclear element
LTR: Long terminal repeat
Mb: Megabase pairs
ML: Maximum likelihood
OBP: Odorant binding protein
OR: Odorant receptor
P450: Cytochrome P450
PCW: Plant cell wall
PL: Polysaccharide lyase
RAxML: Randomized Axelerated Maximum Likelihood
RNAseq: RNA sequencing
SINE: Short interspersed nuclear element
TE: Transposable element
UGT: UDP-glucuronosyltransferase
