# Supplementary figures and images for: The draft genome of the specialist flea beetle Altica viridicyanea (Coleoptera: Chrysomelidae)
Source: BMC Genomics. 2021 Apr 7;22:243. doi: 10.1186/s12864-021-07558-6 (PMC8028732; doi:10.1186/s12864-021-07558-6)

Figure S1

*De novo*

Homolog

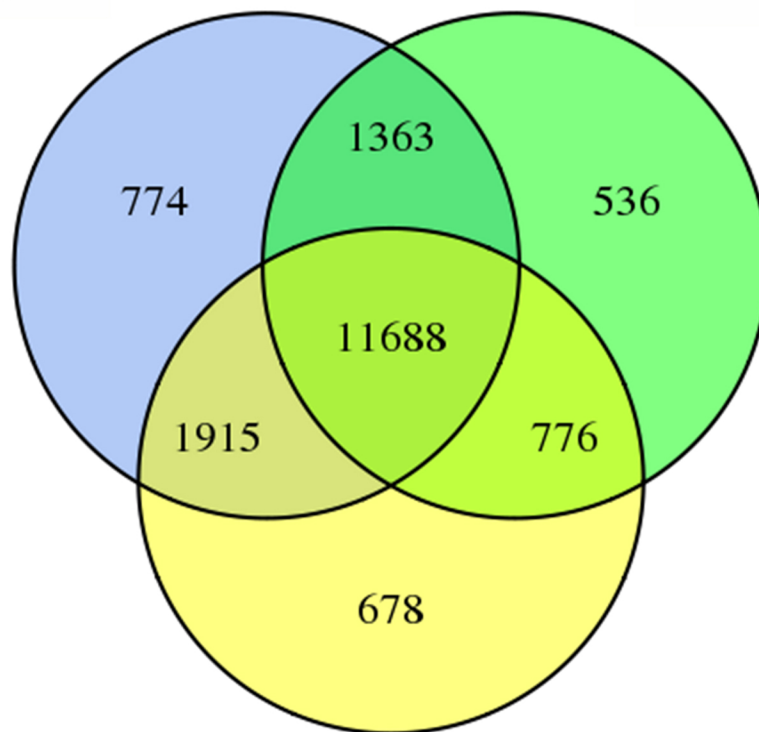

RNAseq

### Figure S2

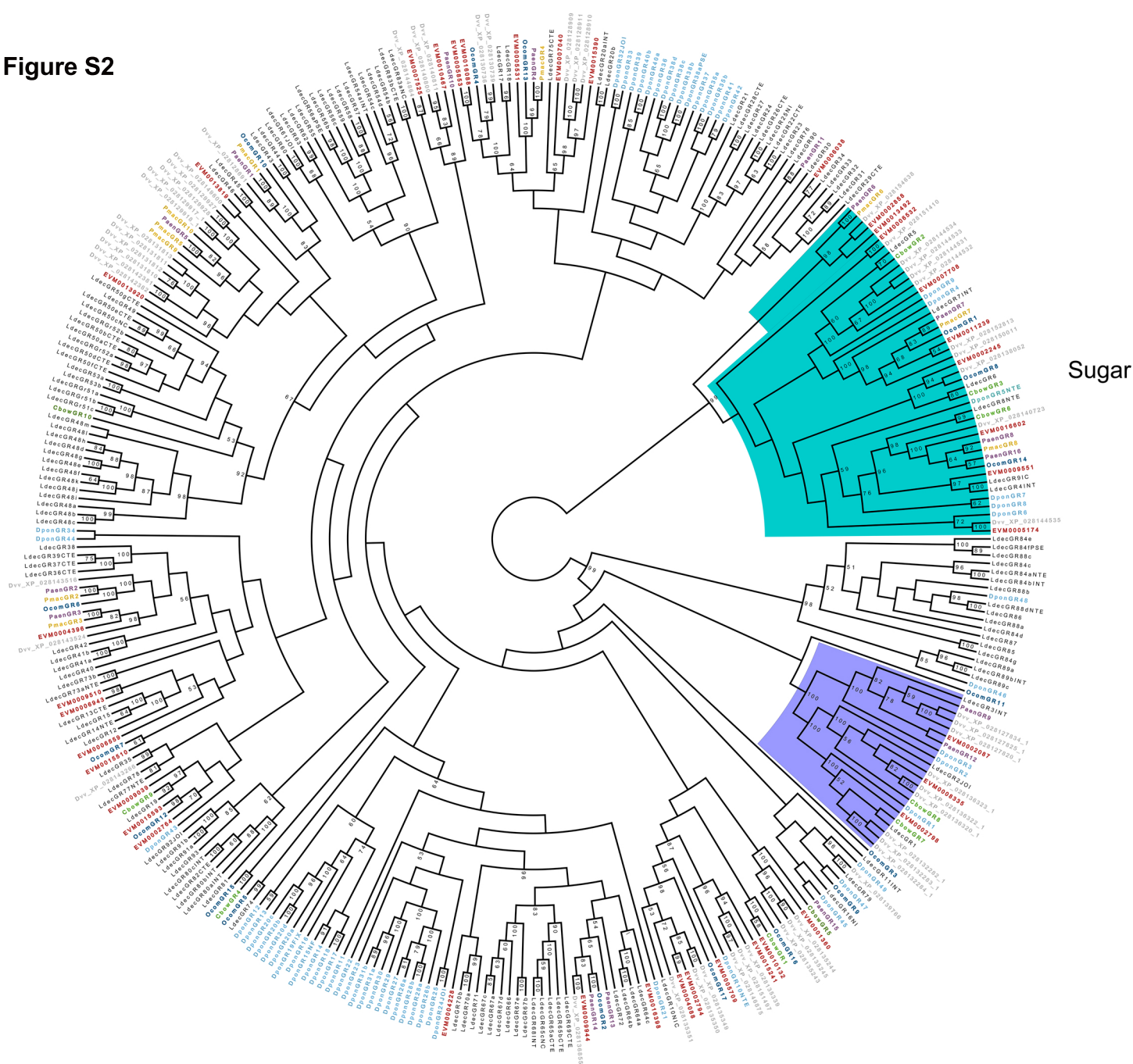

Minus C

Figure S3

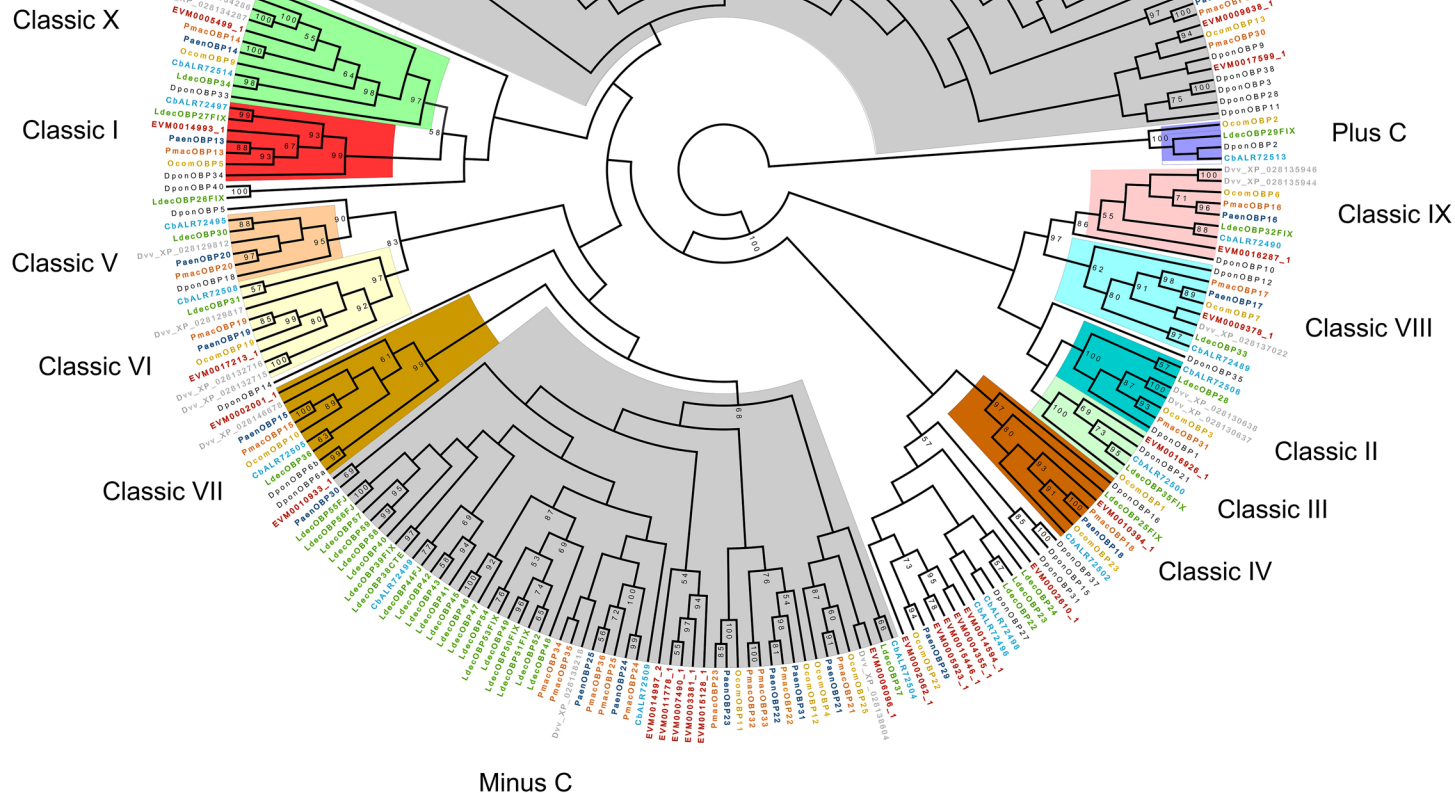

Figure S4

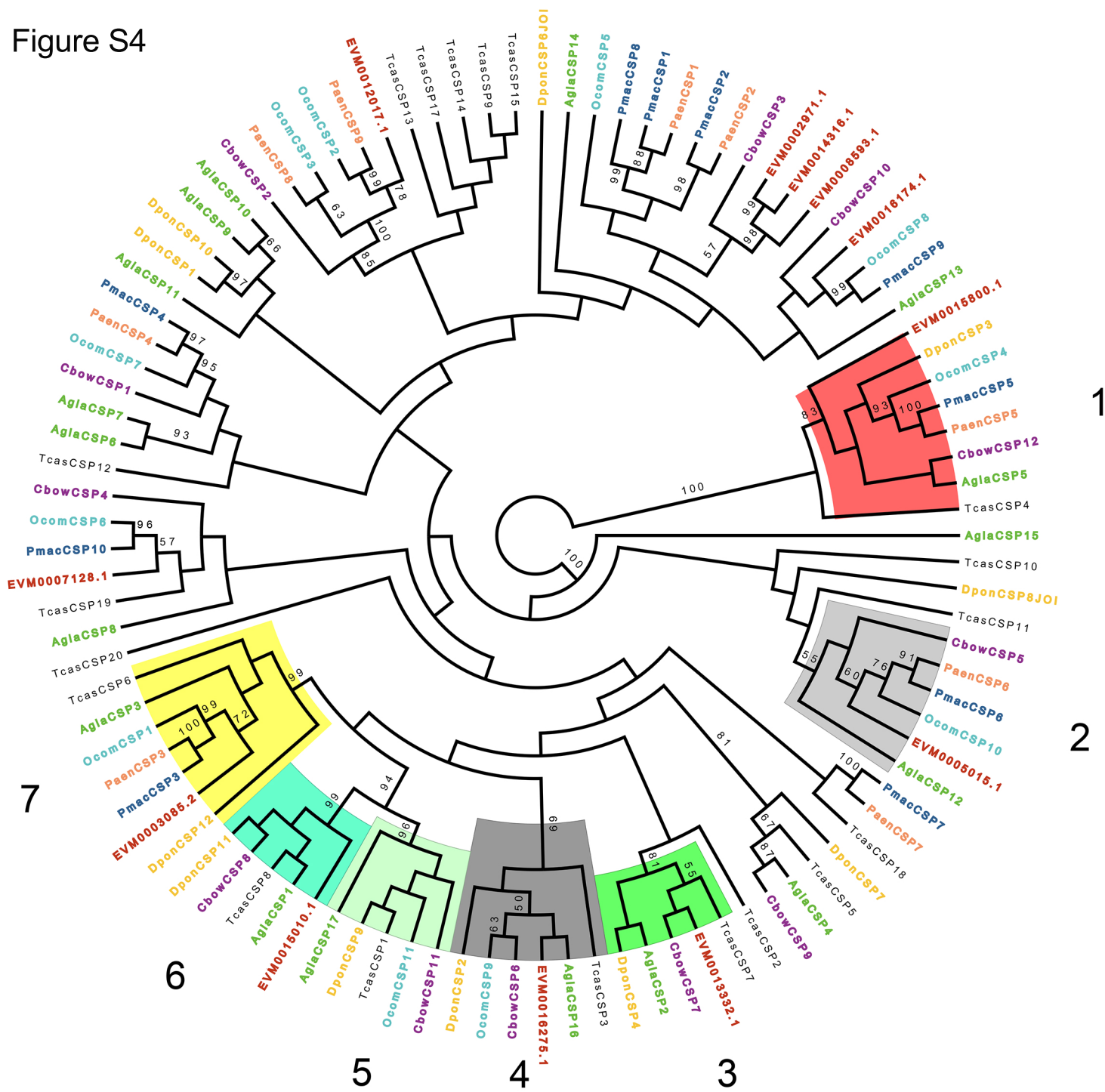

Figure S5

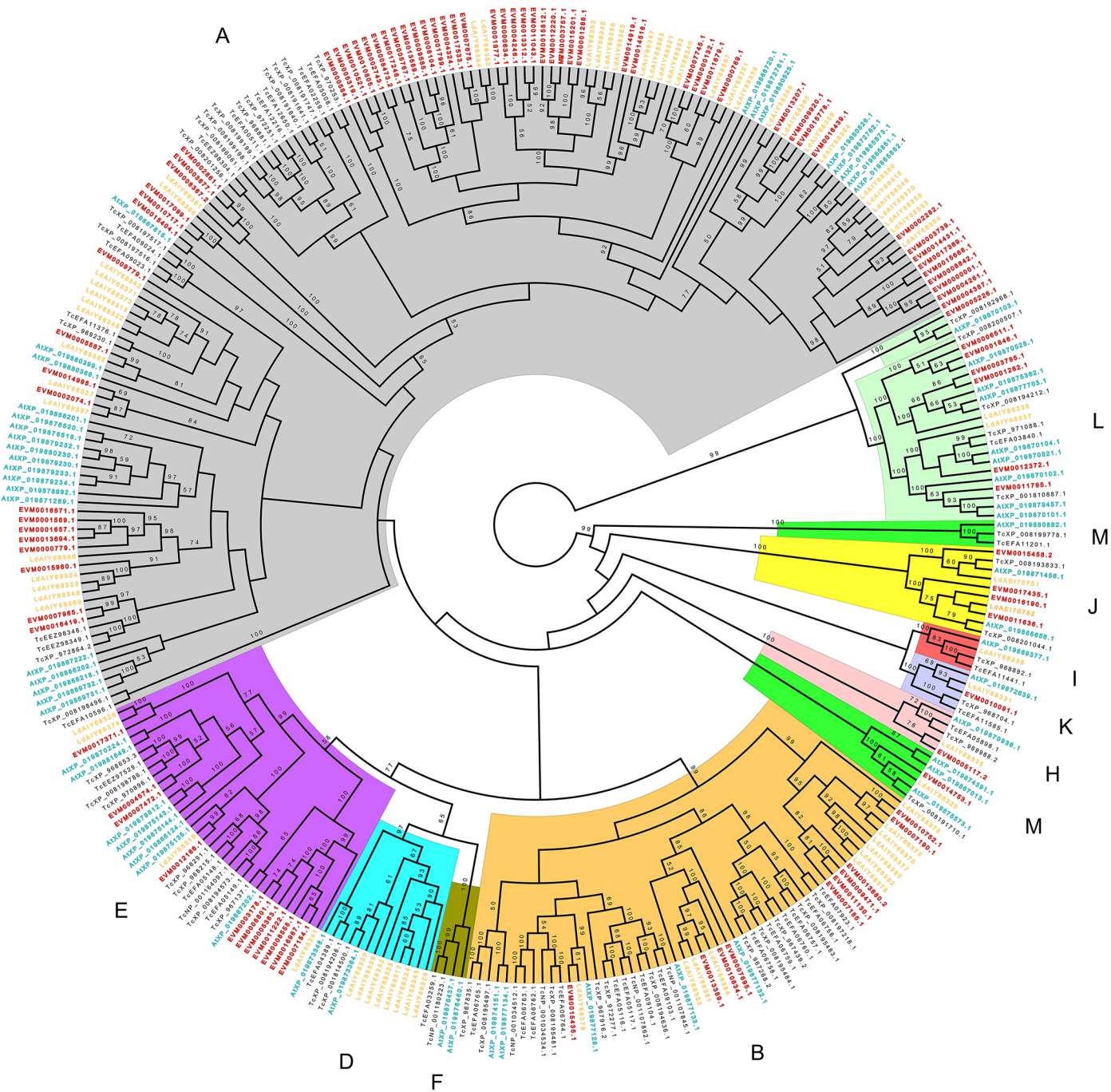

Figure S6

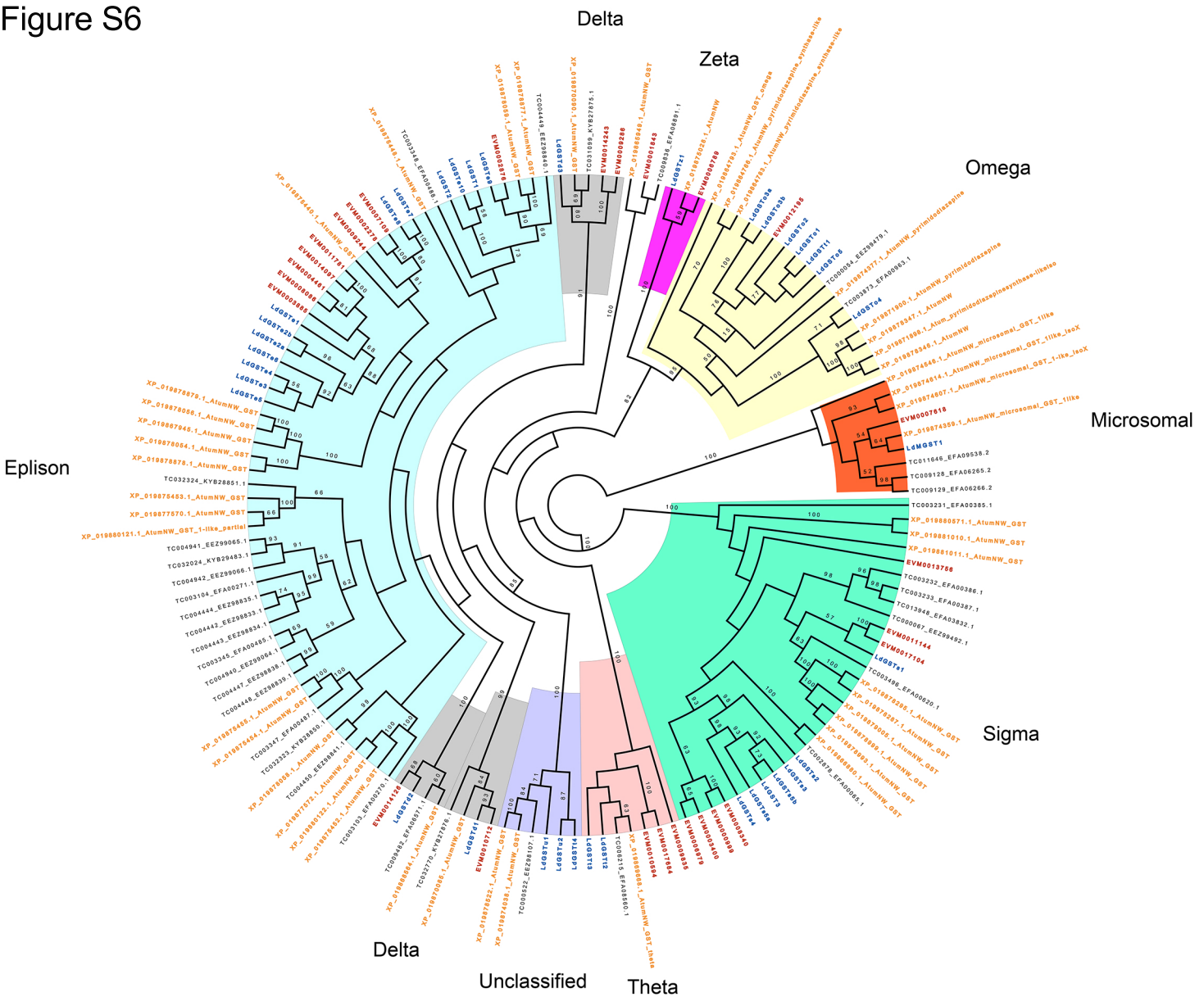

## Figure S7

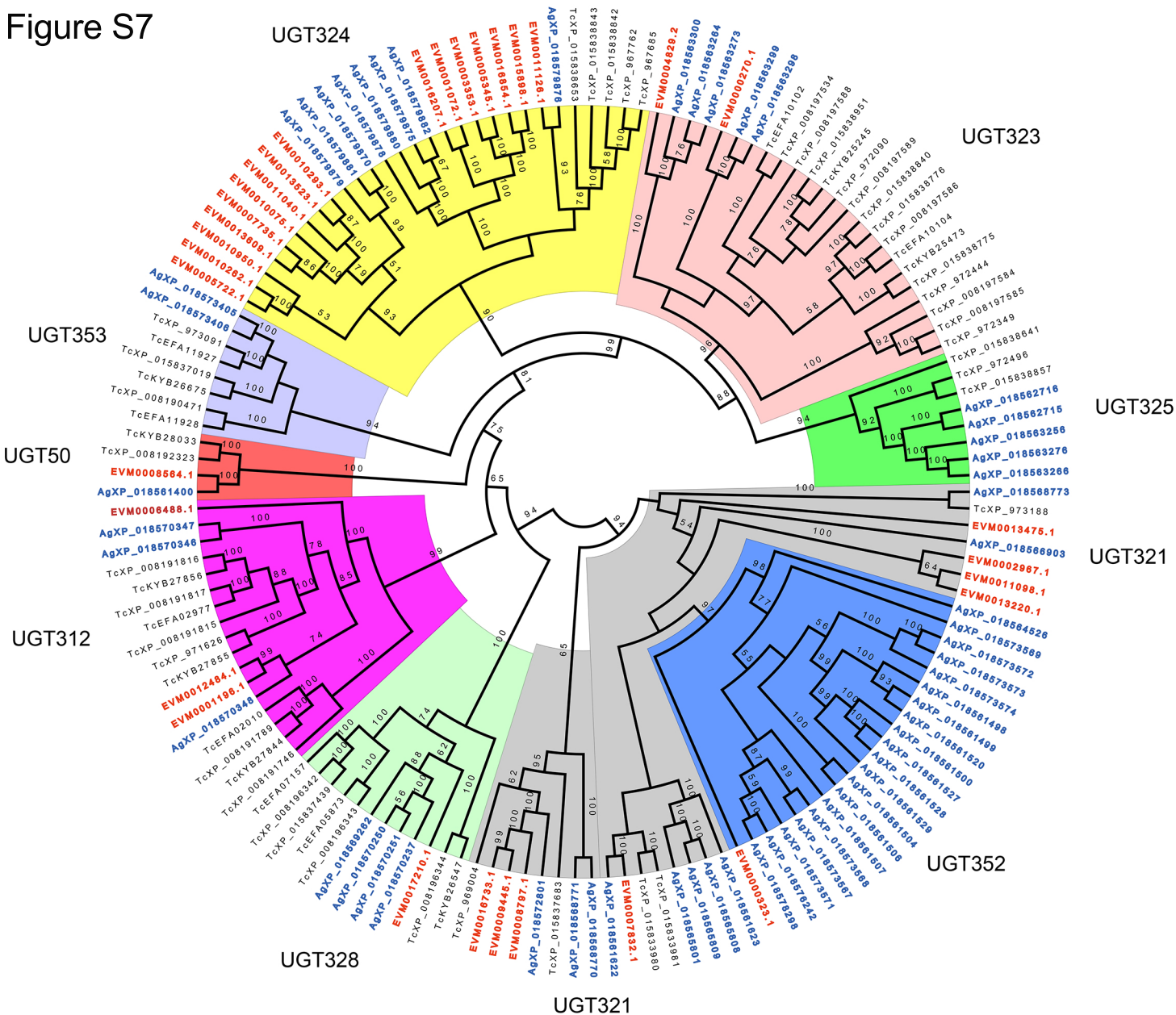

Supplement: Supplementary file 1 — Additional file 1: Figure S1. Venn diagram indicating the number of protein-coding genes of A. viridicyanea based on ab initio, RNA-seq-based and homology-based gene prediction methods. Figure S2. Maximum likelihood cladogram of gustatory receptor genes from eight beetle species. Altica viridicyanea (red labels), Ophraella communa (Ocom, dark blue labels), Colaphellus bowringi (Cbow, green labels), Pyrrhalta aenescens (Paen, purple labels), Pyrrhalta maculicollis (Pmac, yellow labels), Dendroctonus ponderosae (Dpon, pale blue labels), Leptinotarsa decemlineata (Ldec, black labels) and Diabrotica virgifera virgifera (Dvv, grey labels). Node support values lower than 50 are not shown. Figure S3. Maximum likelihood cladogram of odorant binding proteins from eight beetle species. Altica viridicyanea (red labels), Ophraella communa (Ocom, yellow labels), Leptinotarsa decemlineata (Ldec, green labels), Colaphellus bowringi (Cb, pale blue labels), Pyrrhalta aenescens (Paen, dark blue labels), Pyrrhalta maculicollis (Pmac, orange labels), Dendroctonus ponderosae (Dpon, black labels) and Diabrotica virgifera virgifera (Dvv, gray lables). Node support values lower than 50 are not shown. Figure S4. Maximum likelihood cladogram of chemosensory proteins from eight beetle species. Altica viridicyanea (red labels), Ophraella communa (Ocom, pale blue labels), Colaphellus bowringi (Cbow, purple labels), Pyrrhalta aenescens (Paen, orange labels), Pyrrhalta maculicollis (Pmac, dark blue labels), Dendroctonus ponderosae (Dpon, yellow labels), Anoplophora glabripennis (Agla, green labels) and Tribolium castaneum (Tcas, black labels). Node support values lower than 50 are not shown. Figure S5. Maximum likelihood cladogram of carboxyl/cholinesterases from four beetle species. Altica viridicyanea (red labels), Leptinotarsa decemlineata (Ldec, yellow labels), Aethina tumida (At, blue labels) and Tribolium castaneum (Tc, black labels). Node support values lower than 50 are not shown. Figure [file 12864_2021_7558_MOESM1_ESM.pdf]
