# Supplementary material for: The draft genome of the specialist flea beetle Altica viridicyanea (Coleoptera: Chrysomelidae)
Source: BMC Genomics. 2021 Apr 7;22:243. doi: 10.1186/s12864-021-07558-6 (PMC8028732; doi:10.1186/s12864-021-07558-6)
Supplement: Supplementary file 2 — Additional file 2: Table S1. Published beetle genomes. Table S2. Statistics of gene annotation for Altica viridicyanea sourced from different databases. Table S3. Pseudogenes identified in the genome of Altica viridicyanea. Table S4. Gene family classification for the beetle species used in the present study. The incomplete genes were excluded from the analysis. Table S5. Comparison of number of chemosensory functional genes (+pseudogenes) in Altica viridicyanea and other beetle species. Table S6. Comparison of the number of CYP450 genes in Altica viridicyanea and other beetle species. Table S7. Cytochrome P450 genes in Altica viridicyanea. Table S8. Comparison of number of carboxyl/cholinesterases (CCEs) in Altica viridicyanea and other beetle species. Table S9. Comparison of the number of glutathione S-transferases (GSTs) in Altica viridicyanea and other beetle species. Table S10. UDP-glucuronosyltransferases (UGTs) (+pseudogenes) in Altica viridicyanea and other beetle species. Table S11. Comparison of the number of ABC genes in Altica viridicyanea and other beetle species. Table S12. Plant cell wall degrading enzymes identified in Altica viridicyanea and other beetle species. Table S13. Summary of library construction and sequencing of Altica viridicyanea. Table S14. Summary of de novo assemblies of the Altica viridicyanea draft genome. Table S15. The best-fit models for amino acid sequence evolution were selected using the Akaike Information Criterion (AIC) in Prottest v3.4.2. [file 12864_2021_7558_MOESM2_ESM.docx]

**Table S1** Published beetle genomes. “\” means the value is unavailable.

|  | **Beetle species** | **Family** | **Common**  **name** | **Size of genome (Mb)** | **Contig N50 (Kb)** | **Scaffold N50 (Kb)** | **Gene**  **models** | **Repetitive sequence（%）** | **References** |
| --- | --- | --- | --- | --- | --- | --- | --- | --- | --- |
| 1 | *Tribolium castaneum* | Tenebrionidae | red flour beetle | 160 | 73 | 4456.7 | 22611 | ~33 | Tribolium Genome Sequencing Consortium. 2008. |
| 2 | *Dendroctonus ponderosae* | Curculionidae (Scolytinae) | mountain pine beetle | Male/female 202/213 | Male/female 7.5/10.1 | Male/female 597.8/382.1 | Male/female 13088/12873 | Male/female 17/23 | Keeling et al. 2013. |
| 3 | *Hypothenemus hampei* | Curculionidae (Scolytinae) | coffee berry borer | 163 | 10.5 | 44.7 | 19222 | \ | Vega et al. 2015. |
| 4 | *Nicrophorus vespilloides* | Silphidae | burying beetle | 195.3 | 102.1 | 122.4 | 13526 | 12.85 | Cunningham et al. 2015. |
| 5 | *Anoplophora glabripennis* | Cerambycidae | longhorned beetle | 710 | 16.5 | 658.8 | 22035 | \ | McKenna et al. 2016. |
| 6 | *Oryctes borbonicus* | Dynastidae | scarab beetle | 518 | 33.1 | 104.8 | 23278 | 29.2 | Meyer et al. 2016. |
| 7 | *Pyrocoelia*  *pectoralis* | Lampyridae | firefly | 760 | 3040 | \ | 23092 | 44.88 | Fu et al. 2017 |
| 8 | *Leptinotarsa decemlineata* | Chrysomelidae | Colorado potato beetle | 1170 | 4.9 | 414 | 24671 | \ | Schoville et al. 2018. |
| 9 | *Hycleus*  *cichorii* | Meloidae | blister beetle | 111.7 | \ | 79.3 | 13813 | 22.73 | Wu et al. 2018. |
| 10 | *Hycleus phaleratus* | Meloidae | blister beetle | 106.7 | \ | 56.1 | 13725 | 13.47 | Wu et al. 2018. |
| 11 | *Harmonia axyridis* | Coccinellidae | ladybird | 423 | 63.5 | 1600 | \ | \ | Ando et al. 2018 |
| 12 | *Coccinella septempunctata* | Coccinellidae | ladybird | 515 | 67.8 | \ | \ | \ | Ando et al. 2018 |
| 13 | *Photinus*  *pyralis* | Lampyridae | firefly | 471 | 193.4 | 50607 | 15773 | 42.6 | Fallon et al. 2018. |
| 14 | *Aquatica lateralis* | Lampyridae | firefly | 902 | 38 | 690 | 14285 | 19.8 | Fallon et al. 2018. |
| 15 | *Ignelater luminosus* | Elateridae | click beetle | 845 | 31.6 | 116 | 27557 | 34.1 | Fallon et al. 2018. |
| 16 | *Aethina*  *tumida* | Nitidulidae | small hive beetle | 234 | 298.9 | \ | 14076 | \ | Evans et al. 2018. |
| 17 | *Protaetia brevitarsis* | Scarabaeidae | white-spotted flower chafer | 751.1 | \ | 2940 | 34110 | 51.82 | Wang et al. 2019 |
| 18 | *Callosobruchus maculatus* | Chrysomelidae | cowpea weevil | 1007.8 | 212 | \ | 21258 | 64 | Sayadi et al. 2019 |
| 19 | *Aleochara bilineata* | Staphylinidae | rove beetle | 85.9 | 12.1 | 54.1 | 7720 | \ | Kraaijeveld et al. 2019 |
| 20 | *Mylabris*  *aulica* | Meloidae | blister beetle | 288.5 | \ | 467.8 | 16500 | 50.62 | Guan et al. 2020. |
| 21 | *Propylea* *japonica* | Coccinellidae | ladybird | 803.9 | 813.4 | 100340 | 18018 | 71.33 | Zhang et al. 2020 |
| 22 | *Ophraella*  *communa* | Chrysomelidae | ragweed leaf  beetle | 774 | \ | 195.5 | 75642 | 58.2 | Sarah et al. 2020 |

**Table S2** Statistics of gene annotation for *Altica viridicyanea* sourced from different databases.

| **Annotation database** | **Annotated number of genes** | **Percentage (%)** |
| --- | --- | --- |
| GO Annotation | 7,449 | 42.01 |
| KEGG Annotation | 7,601 | 42.87 |
| KOG Annotation | 10,664 | 60.15 |
| TrEMBL Annotation | 16,558 | 93.39 |
| Nr Annotation | 16,533 | 93.25 |
| All Annotated | 16,625 | 93.77 |

**Table** **S3** Pseudogenes identified in the genome of *Altica viridicyanea.*

| **Annotation database** | **Annotated number of pseudogenes** |
| --- | --- |
| GO Annotation | 132 |
| KEGG Annotation | 36 |
| KOG Annotation | 137 |
| TrEMBL Annotation | 743 |
| nr Annotation | 711 |
| All Annotated | 750 |

**Table S4** Gene family classification for the beetle species used in the present study. The incomplete genes were excluded from the analysis.

| **Species name** | **Family** | **Total gene number** | **Cluster gene number** | **Unique gene number** | **Unique gene**  **family number** | **Total family number** |
| --- | --- | --- | --- | --- | --- | --- |
| *Leptinotarsa decemlineata* | Chrysomelidae | 12,949 | 11,134 | 447 | 162 | 9,289 |
| *Anoplophora glabripennis* | Cerambycidae | 14,533 | 13,266 | 505 | 168 | 10,050 |
| *Dendroctonus ponderosae* | Curculionidae | 12,102 | 10,957 | 573 | 213 | 8,972 |
| *Aethina tumida* | Nitidulidae | 13,888 | 12,275 | 712 | 255 | 9,424 |
| *Tribolium castaneum* | Tenebrionidae | 12,841 | 11,713 | 694 | 202 | 9,637 |
| *Onthophagus taurus* | Scarabaeidae | 14,402 | 12,764 | 1,223 | 355 | 9,561 |
| *Nicrophorus vespilloides* | Silphidae | 12,420 | 11,157 | 598 | 147 | 9,305 |
| *Agrilus planipennis* | Buprestidae | 11,373 | 9,940 | 363 | 107 | 8,628 |
| ***Altica viridicyanea*** | **Chrysomelidae** | **17,730** | **15,240** | **1,609** | **529** | **10,173** |

**Table S5** Comparison of number of chemosensory functional genes (+pseudogenes) in *Altica viridicyanea* and other beetle species. “\” means the value is unavailable; “*” means the gene numbers were estimated from transcriptomic data. ORs: Odorant receptors, GRs: Gustatory receptors, IRs: Ionotropic receptors, OBPs: Odorant binding proteins, CSPs: Chemosensory proteins

| **Chemosensory gene subfamily** | **ORs** | **GRs** | **IRs** | **OBPs** | **CSPs** | **Total** | **Reference** |
| --- | --- | --- | --- | --- | --- | --- | --- |
| **Species** |  |  |  |  |  |  |  |
| *Tribolium castaneum* | 264 | 219 | 72 | 49 | \ | 604 | Schoville et al. 2018 |
|  | 270(+68) | 219(+26) | 71(+9) | 50 | 20 | 630(+103) | Andersson et al. 2019 |
| *Dendroctonus ponderosae* | 79(+7) | 59(+1) | 55(+2) | 36 | 11 | 240(+10) | Andersson et al. 2019 |
| *Agrilus planipennis* | 46(+1) | 30 | 30(+1) | 12 | 14 | 132(+2) | Andersson et al. 2019 |
| *Anoplophora glabripennis* | 131  37  121(+11) | 234  11  190(+44) | 72  4  63(+9) | 52  42  60(+1) | \  12  17 | 289  106  451(+65) | McKenna et al. 2016  Hu et al. 2016*  Andersson et al. 2019 |
| *Leptinotarsa decemlineata* | 75  37  76(+4) | 144  \  144(+3) | 27  10  27 | 58  26  58(+1) | \  15  \ | 304  88  305(+8) | Schoville et al. 2018  Liu et al. 2015*  Andersson et al. 2019 |
| *Colaphellus bowringi* | 43 | 10 | 9 | 26 | 12 | 100 | Li et al. 2015* |
| *Pyrrhalta aenescens* | 26 | 16 | 8-3 | 31 | 9 | 90 | Zhang et al. 2016* |
| *Pyrrhalta maculicollis* | 22 | 10 | 7 | 36 | 10 | 85 | Zhang et al. 2016* |
| *Ophraella communa* | 30 | 17 | 18-7 | 25 | 11 | 101 | Ma et al. 2109* |
| *Chrysomela lapponica* | 41 | 8 | 17 | 32 | 12 | 110 | Wang et al. 2018* |
| *Diabrotica virgifera virgifera* | 47 | 54 | 18 | 16 | \ | 135 | https://www.ncbi.nlm.nih.gov/genome/?term=Diabrotica+virgifera |
| ***Altica viridicyanea*** | **63(+1)** | **39** | **12** | **48(+1)** | **12** | **174(+2)** | **Present study** |

**Table S6** Comparison of the number of CYP450 genes in *Altica viridicyanea* and other beetle species. “*” means the gene numbers were estimated from transcriptomic data.

| **P450 clan**  **Beetle species** | **CYP2** | **CYP3** | **CYP4** | **Mitochondrial** | **Total** | **Reference** |
| --- | --- | --- | --- | --- | --- | --- |
| *Oryctes borbonicus* | 6 | 62 | 39 | 8 | 115 | Meyer et al. 2016 |
| *Aethina tumida* | 8 | 55 | 43 | 10 | 116 | Evans et al. 2018 |
| *Tribolium castaneum* | 8 | 82 | 49 | 10 | 149 | Evans et al. 2018 |
| *Photinus pyralis* | 19 | 105 | 34 | 7 | 165 | Fallon et al. 2018 |
| *Dendroctonus ponderosae* | 7 | 47 | 22 | 9 | 85 | Keeling et al. 2013 |
| *Anoplophora glabripennis* | 8 | 63 | 39 | 14 | 124 | McKenna et al. 2016 |
| *Leptinotarsa decemlineata* | 2 | 61 | 28 | 7 | 98  89 | Zhu et al. 2016*  Schoville et al. 2018 |
| ***Altica viridicyanea*** | **7** | **53** | **36** | **5** | **101** | **Present study** |

**Table S7** Cytochrome P450 genes in *Altica viridicyanea*.

| **Clan** | **Subfamily** | **Number** |
| --- | --- | --- |
| Mitochondrial | CYP353 | 1 |
|  | CYP49 | 1 |
|  | CYP12 | 1 |
|  | CYP302 | 1 |
|  | CYP314 | 1 |
| CYP2 | CYP18 | 1 |
|  | CYP305 | 3 |
|  | CYP306 | 1 |
|  | CYP307 | 2 |
| CYP3 | CYP9 | 17 |
|  | CYP6 | 34 |
|  | CYP347 | 2 |
| CYP4 | CYP4 | 36 |
| Total |  | 101 |

**Table S8** Comparison of number of carboxyl/cholinesterases (CCEs) in *Altica viridicyanea* and other beetle species. Clades A and B (dietary), Clade D (integument esterase), Clade E (*β*- and pheromone esterase), Clade F (Juvenile hormone esterase), Clade H (glutactins), Clade I (unknown function), Clade J (acetylcholinesterase), Clade K (gliotactin), Clade L (neuroligin), and Clade M (neurotactin). “*” means the gene numbers were estimated from transcriptomic data.

| **CCE subfamily**  **Beetle species** | **A** | **B** | **D** | **E** | **F** | **H** | **I** | **J** | **K** | **L** | **M** | **Total** | **Reference** |
| --- | --- | --- | --- | --- | --- | --- | --- | --- | --- | --- | --- | --- | --- |
| *Aethina tumida* | 22 | 5 | 2 | 8 | 3 | 2 | 1 | 2 | 1 | 10 | 4 | 60 | Evans et al. 2018 |
| *Tribolium castaneum* | 25 | 29 | 5 | 10 | 1 | 2 | 2 | 2 | 2 | 5 | 1 | 85 | Evans et al. 2018 |
| *Leptinotarsa decemlineata* | 41 | 11 | 8 | 3 | 1 | 1 | 1 | 2 | 2 | 2 | 1 | 72 | Lü et al. 2015* |
|  |  |  |  |  |  |  |  |  |  |  |  | 102 | Rane et al. 2019 |
| ***Altica viridicyanea*** | **62** | **10** | **0** | **11** | **0** | **1** | **0** | **4** | **1** | **7** | **1** | **97** | **Present study** |

**Table S9** Comparison of the number of glutathione S-transferases (GSTs) in *Altica viridicyanea* and other beetle species. “\” means the value is unavailable.

| **GST class**  **Beetle species** | **Delta + Epsilon** | **Omega** | **Sigma** | **Theta** | **Zeta** | **Microsomal** | **Unclassified** | **Total** | **Reference** |
| --- | --- | --- | --- | --- | --- | --- | --- | --- | --- |
| *Oryctes borbonicus* | 8 | 3 | 15 | 3 | 1 | \ | 0 | 30 | Meyer et al. 2016 |
| *Aethina tumida* | 3+19 | 1 | 7 | 1 | 5 | 6 | 7 | 49 | Evans et al. 2018 |
| *Tribolium castaneum* | 3+19 | 3 | 7 | 1 | 1 | 5 | 2 | 41 | Shi et al. 2012 |
| *Dendroctonus ponderosae* | 6+12 | 2 | 5 | 2 | 1 | \ | 0 | 28 | Keeling et al. 2013 |
| *Leptinotarsa decemlineata* | 5+11  3+10 | 4  5 | 3  4 | 2  4 | 0  1 | 2  1 | 0  2 | 27  30 | Schoville et al. 2018  Han et al. 2016* |
| ***Altica viridicyanea*** | **4+9** | **1** | **7** | **3** | **1** | **1** | **0** | **27** | **Present study** |

**Table S10** UDP-glucuronosyltransferases (UGTs) (+pseudogenes) in *Altica viridicyanea* and other beetle species.

| **UGT family**  **Beetle species** | **UGT352** | **UGT321** | **UGT328** | **UGT324** | **UGT323** | **UGT325** | **UGT312** | **UGT353** | **UGT50** | **Total** | **Reference** |
| --- | --- | --- | --- | --- | --- | --- | --- | --- | --- | --- | --- |
| *Tribolium castaneum* | 0 | 6 | 7 | 8 | 12 | 3 | 3 | 3 | 1 | 43 | Ahn et al. 2012 |
| *Anoplophora glabripennis* | 16 (+5) | 13 | 4 | 10 (+1) | 4 | 4 (+1) | 4 | 2 | 1 | 58 (+7) | McKenna et al. 2016 |
| ***Altica viridicyanea*** | **0** | **9** | **1** | **16** | **2** | **0** | **3** | **0** | **1** | **32** | **Present study** |

**Table S11** Comparison of the number of ABC genes in *Altica viridicyanea* and other beetle species. “*” means the gene numbers were estimated from transcriptomic data.

| **ABC subfamily**  **Beetle species** | **A** | **B** | **C** | **D** | **E** | **F** | **G** | **H** | **Total** | **Reference** |
| --- | --- | --- | --- | --- | --- | --- | --- | --- | --- | --- |
| *Aethina tumida* | 4 | 6 | 24 | 2 | 1 | 3 | 13 | 3 | 56  83 | Evans et al. 2018  Rane et al. 2019 |
| *Tribolium castaneum* | 10 | 6 | 35 | 2 | 1 | 3 | 14 | 3 | 74  91 | Evans et al. 2018  Rane et al. 2019 |
| *Chrysomela populi* | 5 | 8 | 29 | 2 | 1 | 3 | 14 | 3 | 65 | Strauss et al. 2014* |
| *Diabrotica virgifera virgifera* | 4 | 7 | 32 | 2 | 1 | 3 | 12 | 4 | 65 | Adedipe et al. 2019* |
| ***Altica viridicyanea*** | **8** | **9** | **37** | **2** | **1** | **3** | **8** | **1** | **69** | **Present Study** |

**Table S12** Plant cell wall degrading enzymes identified in *Altica viridicyanea* and other beetle species. “*” means the gene numbers were estimated from transcriptomic data.

| **Beetle species** | **Gene family** | | | | | | | | | **Total** | **Reference** |
| --- | --- | --- | --- | --- | --- | --- | --- | --- | --- | --- | --- |
|  | **Cellulose/hemicellulose degradation** | | | | |  | **Invertase degradation** |  | **Pectin degradation** |  |  |
|  | **GH1** | **GH5** | **GH9** | **GH45** | **GH48** |  | **GH32** |  | **GH28** |  |  |
| *Nicrophorus vespilloides* | 8 | 0 | 1 | 0 | 0 |  | 0 |  | 0 | 9 | Evans et al. 2018 |
| *Oryctes borbonicus* | 2 | 0 | 1 | 0 | 0 |  | 0 |  | 0 | 3 | Evans et al. 2018 |
| *Tribolium castaneum* | 15 | 0 | 2 | 0 | 0 |  | 0 |  | 0 | 17 | Schoville et al. 2018 |
| *Aethina tumida* | 8 | 0 | 0 | 0 | 0 |  | 0 |  | 0 | 8 | Evans et al. 2018 |
| *Hypothenemus hampei* | 21 | 2 | 0 | 3 | 4 |  | 0 |  | 7 | 37 | Evans et al. 2018 |
| *Dendroctonus ponderosae* | 25  19 | 2  0 | 0  0 | 9  9 | 8  6 |  | 2  3 |  | 23  19 | 69  56 | Evans et al. 2018  McKenna et al. 2019* |
| *Anoplophora* *glabripennis* | 57 | 6 | 1 | 2 | 2 |  | 2 |  | 18 | 88 | McKenna et al. 2016 |
| *Mastostethus salvini* | 135 | 0 | 6 | 0 | 0 |  | 2 |  | 0 | 143 | McKenna et al. 2019* |
| *Oreina cacaliae* | 25 | 0 | 1 | 12 | 3 |  | 0 |  | 10 | 51 | McKenna et al. 2019* |
| *Leptinotarsa decemlineata* | 49 | 0 | 0 | 13 | 2 |  | 0 |  | 15 | 79 | Schoville et al. 2018 |
| *Diabrotica undecimpunctata* | 228 | 0 | 0 | 53 | 16 |  | 0 |  | 51 | 348 | McKenna et al. 2019* |
| ***Altica viridicyanea*** | **35** | **0** | **0** | **10** | **2** |  | **0** |  | **18** | **65** | **Present study** |

**Table S13** Summary of library construction and sequencing of *Altica viridicyanea*.

|  |  |  | **Raw data** | | | **Clear data** | | |
| --- | --- | --- | --- | --- | --- | --- | --- | --- |
| **Platform** | **Library** | **Beetle used** | **Raw base (G)** | **Raw reads (M)** | **Sequencing depth (×)** | **Clean base (G)** | **Clean reads** | **Sequencing depth (×)** |
|  |  |  |  |  |  |  |  |  |
| **PacBio** | 170111 | 10 | 15.2 | 2.1 | 18.31 |  |  |  |
|  | 171224 | 30 | 45.2 | 4.6 | 54.46 |  |  |  |
| **Total** |  | 40 | 60.4 | 6.7 | 72.77 |  |  |  |
|  |  |  |  |  |  |  |  |  |
| **Illumina** | ins_270 |  | 44.1 | 293.6 | 55.47 | 43.8 | 292.9 | 55.09 |
|  | ins_500 |  | 24.4 | 162.8 | 29.40 | 24.3 | 162.5 | 29.28 |
|  | ins_800 |  | 15.8 | 105.4 | 19.04 | 15.7 | 105.3 | 18.92 |
|  | ins_3k |  | 10.4 | 91.8 | 12.53 | 10.3 | 91.7 | 12.41 |
|  | ins_5k-1 |  | 29.8 | 198.7 | 35.90 | 21.1 | 163.8 | 25.42 |
|  | ins_5k-2 |  | 11.5 | 101.2 | 13.86 | 11.4 | 100.9 | 13.73 |
|  | ins_10k |  | 17.5 | 116.8 | 21.08 | 12.2 | 95.5 | 14.70 |
| **Total** | - | 117 | 153.5 | 1070.3 | 187.28 | 138.8 | 1012.6 | 169.55 |

**Table S14** Summary of *de novo* assemblies of the *Altica viridicyanea* draft genome.

| Contig (assembled using Flye)  PacBio sequences | Size including N | 842,858,754 |
| --- | --- | --- |
|  | Size excluding N | 842,858,754 |
|  | Number of Sequences | 17580 |
|  | Mean Size | 47,944 |
|  | Median Size | 25,036 |
|  | Longest Sequence | 1,350,991 |
|  | GC Content | 31.67% |
|  | N50 | 92,835 |
|  | L50 | 2,472 |
|  | N90 | 23,835 |
|  | Gap | 0 |
| Scaffold (assembled using Redundans)  Illumina sequences | Size including N | 850,610,146 |
|  | Size excluding N | 843,773,239 |
|  | Number of Sequences | 8,496 |
|  | Mean Size | 100,118 |
|  | Median Size | 23,145 |
|  | Longest Sequence | 5,013,415 |
|  | GC Content | 31.67% |
|  | N50 | 353,225 |
|  | L50 | 611 |
|  | N90 | 53,898 |
|  | Gap | 0.80% |
| Re-scaffold (assembled using SSPACE_long)  PacBio sequences | Size including N | 864,763,381 |
|  | Size excluding N | 844,332,740 |
|  | Number of Sequences | 4,490 |
|  | Mean Size | 192,597 |
|  | Median Size | 58,516 |
|  | Longest Sequence | 5,674,939 |
|  | GC Content | 31.67% |
|  | N50 | 557,265 |
|  | L50 | 429 |
|  | N90 | 122,770 |
|  | Gap | 2.36% |

**Table S15** The best-fit models for amino acid sequence evolution were selected using the Akaike Information Criterion (AIC) in Prottest v3.4.2.

| Chemosensory genes | Best-fit model | Detoxification genes | Best-fit model |
| --- | --- | --- | --- |
| CSP | LG+I+G | GST | LG+I+G+F |
| GR | JTT+G+F | CCE | WAG+I+G+F |
| IR | WAG+ G+F | P450 | LG+G+F |
| OBP | LG+I+G | UGT | LG+I+G+F |
| OR | JTT+G+F | ABC | LG+I+G+F |

**References**

Adedipe F, Grubbs N, Coates B, Wiegmman B, Lorenzen M. 2019. Structural and functional insights into the *Diabrotica virgifera virgifera* ATP-binding cassette transporter gene family. BMC Genomics 20: 899.

Ahn SJ, Vogel H, Heckel DG. 2012. Comparative analysis of the UDP-glycosyltransferase multigene family in insects. Insect Biochemistry and Molecular Biology 42: 133–147.

Andersson MN, Keeling CI, Mitchell RF. 2019. Genomic content of chemosensory genes correlates with host range in wood-boring beetles (*Dendroctonus ponderosae, Agrilus planipennis*, and *Anoplophora glabripennis*). BMC Genomics 20: 690.

Ando T, Matsuda T, Goto K, Hara K, Ito A, Hirata J, Yatomi J, Kajitani R, Okuno M, Yamaguchi K, Kobayashi M, Takano T, Minakuchi Y, Seki M, Suzuki Y, Yano K, Itoh T, Shigenobu S, Toyoda A, Niimi T. 2018. Repeated inversions within a pannier intron drive diversification of intraspecific colour patterns of ladybird beetles. Nature Communications 9: 3843.

Cunningham CB, Ji LX, Wiberg RAW, Shelton J, McKinney EC, Parker DJ, Meagher RB, Benowitz KM, Roy-Zokan EM, Ritchie MG, Brown SJ, Schmitz RJ, Moore AJ. 2015. The genome and methylome of a beetle with complex social behavior, *Nicrophorus vespilloides* (Coleoptera: Silphidae). Genome Biology and Evolution 7: 3383–3396.

Evans JD, McKenna D, Scully E, Cook SC, Dainat B, Egekwu N, Grubbs N, Lopez D, Lorenzen MD, Reyna SM, Rinkevich FD, Neumann P, Huang Q. 2018. Genome of the small hive beetle (*Aethina tumida*, Coleoptera: Nitidulidae), a worldwide parasite of social bee colonies, provides insights into detoxification and herbivory*.* GigaScience 7: 1–16.

Fallon TR, Lower SE, Chang CH, Bessho-Uehara M, Martin GJ, Bewick AJ, Behringer M, Debat HJ, Wong I, Day JC, Suvorov A, Silva CJ, Stanger-Hall KF, Hall DW, Schmitz RJ, Nelson DR, Lewis SM, Shigenobu S, Bybee SM, Larracuente AM, Oba Y, Weng JK. 2018. Firefly genomes illuminate parallel origins of bioluminescence in beetles. eLife 7:e36495.

Fu XH, Li JJ, Tian Y, Quan WP, Zhang S, Liu Q, Liang F, Zhu XL, Zhang LS, Wang DP, Hu J. 2017. Long-read sequence assembly of the firefly *Pyrocoelia pectoralis* genome. GigaScience 6: 1–7.

Guan DL, Hao XQ, Mi D, Peng J, Li Y, Xie JY, Huang HT, Xu SQ. 2020. Draft genome of a blister beetle Mylabris aulica. Frontiers in Genetics 10: 1281.

Han JB, Li GQ, Wan PJ, Zhu TT, Meng QW. 2016. Identification of glutathione S-transferase genes in *Leptinotarsa decemlineata* and their expression patterns under stress of three insecticides. Pesticide Biochemistry and Physiology 133: 26–34.

Hu P, Wang JZ, Cui MM, Tao J, Luo YQ. 2016. Antennal transcriptome analysis of the Asian longhorned beetle *Anoplophora glabripennis*. Scientific Reports 6: 26652.

Keeling CI, Yuen MMS, Liao NY, Docking T R, Chan S K, Taylor GA, Palmquist DL, Jackman SD, Nguyen A, Li M, Henderson H, Janes JK, Zhao YJ, Pandoh P, Moore R, Sperling FAH, Huber DP W, Birol I, Jones SJM, Bohlmann J. 2013. Draft genome of the mountain pine beetle, *Dendroctonus ponderosae* Hopkins, a major forest pest. Genome Biology 14: R27.

Keeling CI, Yuen MMS, Liao NY, Docking T R, Chan S K, Taylor GA, Palmquist DL, Jackman SD, Nguyen A, Li M, Henderson H, Janes JK, Zhao YJ, Pandoh P, Moore R, Sperling FAH, Huber DP W, Birol I, Jones SJM, Bohlmann J. 2013. Draft genome of the mountain pine beetle, *Dendroctonus ponderosae* Hopkins, a major forest pest. Genome Biology 14: R27.

Kraaijeveld K, Neleman P, Mariën J, de Meijer E, Ellers J. 2019. Genomic resources for *Goniozus legneri*, *Aleochara bilineata* and *Paykullia maculata*, representing three independent origins of the parasitoid lifestyle in insects. G3-Genes Genomes Genetics 9: 987–991.

Li XM, Zhu XY, Wang ZQ, Wang Y, He P, Chen G, Sun L, Deng DG, Zhang YN. 2015. Candidate chemosensory genes identified in *Colaphellus bowringi* by antennal transcriptome analysis. BMC Genomics 16: 1028.

Liu Y, Sun LJ, Cao DP, Walker WB, Zhang YQ, Wang GR. 2015. Identification of candidate olfactory genes in *Leptinotarsa decemlineata* by antennal transcriptome analysis. Frontiers in Ecology and Evolution 3: 60.

Liu Y, Sun LJ, Cao DP, Walker WB, Zhang YQ, Wang GR. 2015. Identification of candidate olfactory genes in *Leptinotarsa decemlineata* by antennal transcriptome analysis. Frontiers in Ecology and Evolution 3: 60.

Lü FG, Fu KY, Li Q, Guo WC, Ahmat T, Li GQ. 2015. Identification of carboxylesterase genes and their expression profiles in the Colorado potato beetle *Leptinotarsa decemlineata* treated with fipronil and cyhalothrin. [Pesticide Biochemistry and Physiology](http://www.sciencedirect.com/science/journal/00483575) 122: 86–95.

Ma C, Zhao CC, Cui SW, Zhang Y, Chen GM, Chen HS, Wan FH, Zhou ZS. 2019. Identification of candidate chemosensory genes of *Ophraella communa* LeSage (Coleoptera: Chrysomelidae) based on antennal transcriptome analysis. Scientific Reports 9: 15551.

McKenna DD, Scully ED, Pauchet Y, Hoover K, Kirsch R, Geib SM, Mitchell RF, Waterhouse RM, Ahn SJ, Arsala D, Benoit JB, Blackmon H, Bledsoe T, Bowsher JH, Busch A, Calla B, Chao H, Childers AK, Childers C, Clarke DJ, Cohen L, Demuth JP, Dinh H, Doddapaneni H, Dolan A, Duan JJ, Dugan S, Friedrich M, Glastad KM, Goodisman MA, Haddad S, Han Y, Hughes DS, Ioannidis P, Johnston JS, Jones JW, Kuhn LA, Lance DR, Lee CY, Lee SL, Lin H, Lynch JA, Moczek AP, Murali SC, Muzny DM, Nelson DR, Palli SR, Panfilio KA, Pers D, Poelchau MF, Quan H, Qu J, Ray AM, Rinehart JP, Robertson HM, Roehrdanz R, Rosendale AJ, Shin S, Silva C, Torson AS, Jentzsch IM, Werren JH, Worley KC, Yocum G, Zdobnov EM, Gibbs RA, Richards S. 2016. Genome of the Asian longhorned beetle (*Anoplophora glabripennis*), a globally significant invasive species, reveals key functional and evolutionary innovations at the beetle-plant interface. Genome Biology 17: 227.

McKenna DD, Shin S, Ahrens D, Balke M, Beza-Beza C, Clarke DJ, Donath A, Escalona HE, Friedrich F, Letsch H, Liu SL, Maddison D, Mayer C, Misof B, Murin PJ, Niehuis O, Peters RS, Podsiadlowski L, Pohl H, Scully ED, Yan EV, Zhou X, Ślipiński A, Beutel RG. 2019. The evolution and genomic basis of beetle diversity. Proceedings of the National Academy of Sciences of the United States of America 116: 24729–24737.

Meyer JM, Markov GV, Baskaran P, Herrmann M, Sommer RJ, Rödelsperger C. 2016. Draft genome of the scarab beetle *Oryctes borbonicus* on La Réunion Island. Genome Biology and Evolution 8: 2093–2105.

Rane RV, Ghodke AB, Hoffmann AA, Edwards OR, Walsh TK, Oakeshott JG. 2019. Detoxifying enzyme complements and host use phenotypes in 160 insect species. Current Opinion in Insect Science 31:131–138.

Sarah B, Laurent F, Heinz M-S. 2020. Genome assembly of the ragweed leaf beetle: a step forward to better predict rapid evolution of a weed biocontrol agent to environmental novelties. Genome Biology and Evolution <https://doi.org/10.1093/gbe/evaa102>.

Sayadi A, Barrio AM, Immonen E, Dainat J, Berger D, Tellgren-Roth C, Nystedt B, Arnqvist G. 2019. The genomic footprint of sexual conflict. Nature Ecology & Evolution 3: 1725–1730.

Schoville SD, Chen YH, Andersson MN, Benoit JB, Bhandari A, Bowsher JH, Brevik K, Cappelle K, Chen MJM, Childers AK, Childers C, Christiaens O, Clements J, Didion EM, Elpidina EN, Engsontia P, Friedrich M, García-Robles I, Gibbs RA, Goswami C, Grapputo A, Gruden K, Grynberg M, Henrissat B, Jennings EC, Jones JW, Kalsi M, Khan SA, Kumar A, Li F, Lombard V, Ma XZ, Martynov A, Miller NJ, Mitchell RF, Munoz-Torres M, Muszewska A, Oppert B, Palli SR, Panfilio KA, Pauchet Y, Perkin LC, Petek M, Poelchau MF, Record É, Rinehart JP, Robertson HM, Rosendale AJ, Ruiz-Arroyo VM, Smagghe G, Szendrei Z, Thomas GWC, Torson AS, Jentzsch IMV, Weirauch MT, ™Yates AD, Yocum GD, Yoon JS, Richards S. 2018. A model species for agricultural pest genomics: the genome of the Colorado potato beetle, *Leptinotarsa decemlineata* (Coleoptera: Chrysomelidae). Scientific Reports 8:1931.

Shi HX, Pei LH, Gu SS, Zhu SC, Wang YY, Zhang Y, Li B. 2012. Glutathione S-transferase (GST) genes in the red flour beetle, *Tribolium castaneum*, and comparative analysis with five additional insects. Genomics 100: 327–335.

Strauss AS, Wang D, Stock M, Gretscher RR, Groth M, Boland W, Burse A. 2014. Tissue-specific transcript profiling for ABC transporters in the sequestering larvae of the phytophagous leaf beetle *Chrysomela populi*. PLoS ONE 9: e98637.

Tribolium Genome Sequencing Consortium. 2008. The genome of the model beetle and pest *Tribolium castaneum*. Nature 452: 949–955.

Vega FE, Brown SM, Chen H, Shen E, Nair MB, Ceja-Navarro JA, Brodie EL, Infante F, Dowd PF, Pain A. 2015. Draft genome of the most devastating insect pest of coffe worldwide: the coffee berry borer, *Hypothenemus ham*pei. Scientific Reports 5:12525

Wang D, Pentzold S, Kunert M, Groth M, Brandt W, Pasteels JM, Boland W, Burse A. 2018. A subset of chemosensory genes differs between two populations of a specialized leaf beetle after host plant shift. Ecology and Evolution 8: 8055–8075.

Wang K, Li PP, Gao YY, Liu CQ, Wang QL, Yin J, Zhang J, Geng LL, Shu CL. 2019. De novo genome assembly of the white-spotted flower chafer (*Protaetia brevitarsis*). GigaScience 8: 1–9.

Wu YM, Li J, Chen XS. 2018. Draft genomes of two blister beetles *Hycleus cichorii* and *Hycleus phaleratus*. GigaScience 7: 1–7.

Zhang B, Zhang W, Nie RE, Li WZ, Segraves KA, Yang XK, Xue HJ. 2016. Comparative transcriptome analysis of chemosensory genes in two sister leaf beetles provides insights into chemosensory speciation. Insect Biochemistry and Molecular Biology 79: 108 –118.

Zhang LJ, Li S, Luo JY, Du P, Wu LK, Li YR, Zhu XZ, Wang L, Zhang S, Cui JJ. 2020. Chromosome-level genome assembly of the predator *Propylea japonica* to understand its tolerance to insecticides and high temperatures. Molecular Ecology Resources 20: 292–307.

Zhu F, Moural TW, Nelson DR, Palli SR. 2016. A specialist herbivore pest adaptation to xenobiotics through upregulation of multiple cytochrome P450s. Scientific Reports 6: 20421.
